# Mechanism of regulation of KIF23 on endometrial cancer cell growth and apoptosis

**DOI:** 10.1007/s12672-024-00937-x

**Published:** 2024-03-21

**Authors:** Ruiying Zhuang, Haiyan Liu

**Affiliations:** 1 Jinzhou Medical University, Jinzhou, Liaoning Province China; 2 The First Affiliated Hospital of Jinzhou Medical University, Jinzhou, Liaoning Province China

**Keywords:** KIF23, Endometrial cancer, Cell death, Cell proliferation

## Abstract

**Objective:**

The global incidence of endometrial cancer, a malignant tumor in females, is on the rise. It is one of the most common gynecological cancers. Early-stage endometrial cancers can often be treated successfully with uterine extirpation. However, those diagnosed at a later stage have a poor prognosis and encounter treatment challenges. Therefore, additional research is necessary to develop primary prevention strategies for high-risk women and improve survival rates among patients with endometrial cancer. Hence, gene therapy targeting KIF23 shows promise as an advanced strategy for the treatment of endometrial cancer.

**Methods:**

Immunohistochemistry, Western blotting, and PCR were used to examine the expression of KIF23 and its associated pathway factors in endometrial cancer tissue (specifically Ishikawa and SNGM cells, respectively). We investigated the functional roles of KIF23 using CCK-8, colony-forming proliferation assays, Transwell migration assays, and xenotransplantation in mice.

**Results:**

Immunohistochemistry analysis showed variations in the expression levels of KIF23 between endometrial cancer tissue and normal endometrium tissue. KIF23 downregulated BAX and caspase-3 protein expression while upregulating BCL-2 protein expression. Additionally, knocking out KIF23 inhibits endometrial cancer cell proliferation and migration while promoting cell death. Mechanistically, our study provides evidence that KIF23 promotes endometrial cancer cell proliferation by activating the ERK and AKT/PI3K pathways, while simultaneously inhibiting programmed cell death in endometrial cancer.

**Conclusion:**

Our study provides evidence to support the inhibition of endometrial cancer by KIF23 knockdown. This offers valuable insights for future research on potential therapeutic strategies for this type of cancer.

**Graphical Abstract:**

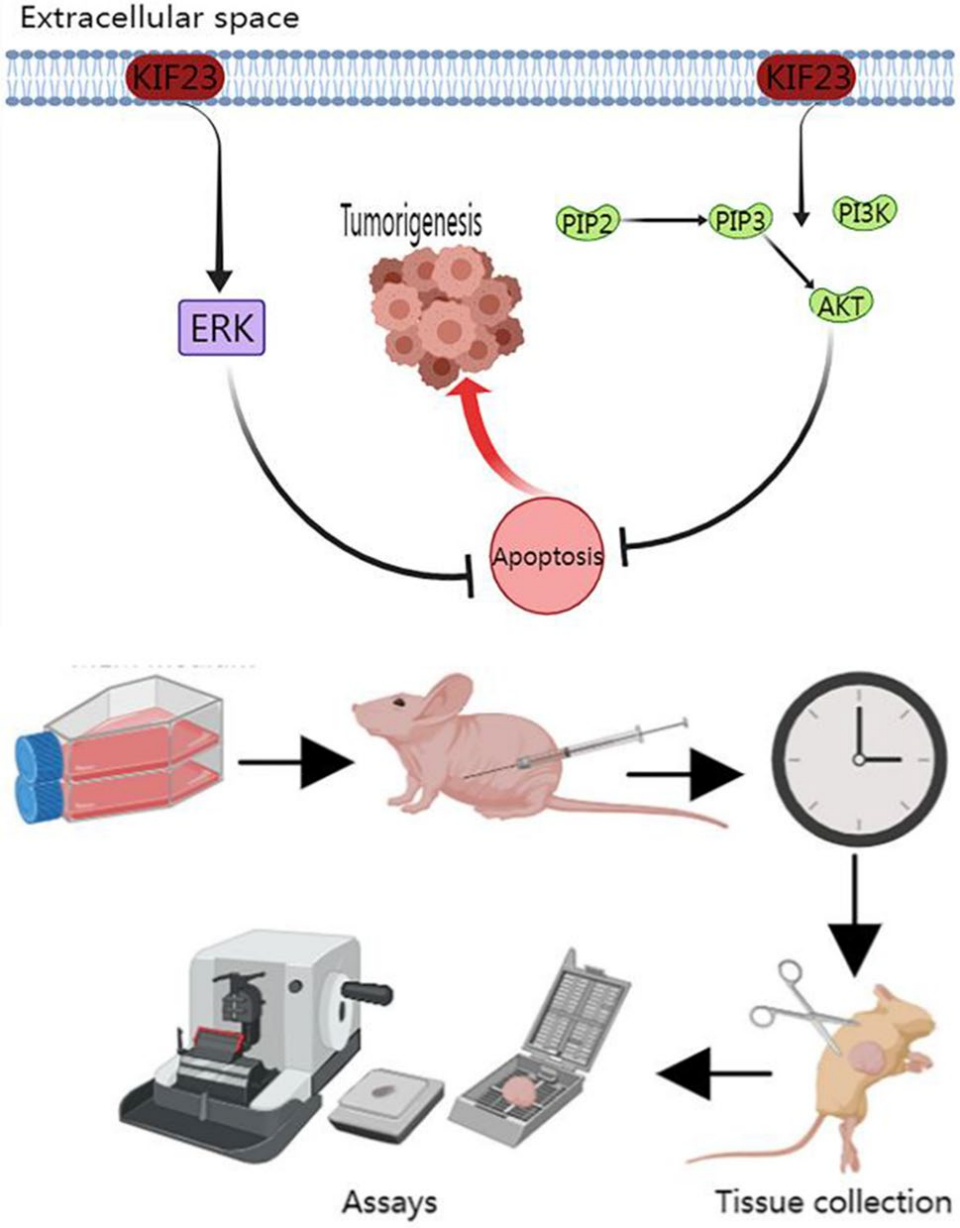

**Supplementary Information:**

The online version contains supplementary material available at 10.1007/s12672-024-00937-x.

## Introduction

Endometrial cancer is one of the most frequently occurring gynecologic malignancies in women.It exhibits a notable upward trend, with an estimated incidence rate of approximately 5.9% [1]. Unfortunately, there has been no significant improvement in the mortality rate associated with this cancer [2]. The primary surgical approach for treating endometrial cancer involves abdominal or laparoscopic hysterectomy and bilateral salpingo-oophorectomy [3]. Nonetheless, these surgeries may affect the fertility of certain younger women [4], emphasizing the importance of individualized adjunctive therapies for different types of endometrial cancer. There is increasing interest in targeted therapies that target the human immune system and cellular signaling pathways. These therapies are garnering attention and can be utilized as either combination or standalone therapies [3]. While numerous clinical trials are exploring molecular-targeted therapies, no approved targeted therapy currently exists for EC. Given the importance of improving outcomes for patients with endometrial cancer, it is crucial to identify novel target genes, elucidate the molecular mechanisms underlying its development, and develop innovative targeted therapies. The emergence of gene therapy has opened up entirely new treatment possibilities for patients with malignancies.

The mitotic kinesin-like protein family, also known as KIF23 or MKLP1, are microtubule-associated proteins that are located at the spindle midzone [5]. These proteins were first identified by Nislow et al. in 1992 [6]. The Kinesin family (KIF) is a group of molecular motor proteins that are characterized by having ATP enzyme activity in their spherical heads. As a transporter in cells, KIF proteins obtain the required energy by hydrolyzing ATP, which allows them to change their configuration [7]. KIF proteins can directionally transport various cargos along microtubules [7, 8]. KIF proteins not only mediate cellular processes [9], but are also involved in cell division, the transport of intracellular vesicles and organelles [10–12], cytoskeleton organization, ciliary function [13, 14], chromosome and RNA-binding protein transport [15–17], as well as spindle and chromosome separation intermediates [18, 19]. KIF23 plays a crucial role in filament division [20].

Abnormal expression of KIF23 can affect cytokinesis and centrosome formation, leading to cell division stagnation or abnormality [21, 22]. Abnormal cytokinesis may lead to cell death, while surviving cells can exhibit genomic instability [23–25]. The formation of binuclear or polynuclear cells can contribute to cancer development [26, 27].

Because of its specific role in filament separation, KIF23 may have fewer side effects compared to other agents used in anticancer treatment [28]. A growing body of evidence suggests that KIF23 may play a role in tumorigenesis. This emphasizes the importance of further investigating the role of KIF23 in endometrial cancer.

We silenced KIF23 in Ishikawa and SNGM endometrial cancer cell lines to investigate its effects on tumor growth. Subsequently, we developed a nude mouse model for the heterogeneous transplantation of endometrial cancer to validate the inhibitory effect of KIF23 on endometrial cancer development in vivo. Our research aims to understand the functional effectiveness of KIF23 as a therapeutic agent for endometrial cancer, thereby laying the groundwork for its potential application in EC therapy (Additional fle 1).

## Materials and methods

### GEPIA database

The GEPIA online database offers quick and customizable analytical capabilities based on TCGA and GTEx data.GEPIA's features include differential expression analysis, gene mapping, correlation analysis, and analysis of patient survival rates and survival periods. We accessed the GEPIA database, entered KIF23 into the search field, clicked on Expression DIY (Boxplot), and selected UCEC (cancerous uterine corpus endometrial carcinoma) to analyze the differential expression of KIF23 in normal tissue and endometrial cancer.

### Cell lines

The Ishikawa cell line is a well-differentiated endometrial adenocarcinoma cell line derived from human endometrial cancer cells with an adherent epithelioid phenotype. Nishida et al., a Japanese scientist, initially discovered and established this cell line in 1985. It has since been widely utilized in endometrial cancer research. The SNGM cell line, derived from human endometrial adenocarcinoma, features spindle- or polygonal-shaped cells with an epithelial-like structure.

### Endometrial cancer samples

From 2018 to 2020, Jinzhou Medical University's First Affiliate Hospital in China collected a total of 68 samples of endometrial cancer tissue and 59 samples of normal endometrial tissue. All patients enrolled in the study provided informed consent and had not undergone chemotherapy or radiation therapy prior to surgery. This research has been approved by the Ethics Committee of the First Affiliated Hospital of Jinzhou Medical University.

### Cell culture

We purchased human endometrial cancer cells (Ishikawa and SNGM) from Shanghai MingJin Cell Biology Co., Ltd. To cultivate Ishikawa cells, we used MEM medium containing NEAA (Pricella, China), while F-12 K medium (Pricella, China) was used for SNGM cells. We added 10% fetal bovine serum (VivaCell, USA) and penicillin–streptomycin (100 g/mL) (Servicebio, China) to the medium. For cell subculture, we used trypsin–EDTA solution with phenol red (Wanleibio, China). Cells were cultured at 37 °C with 5% CO2.

### Cell transfection

To alter the expression of KIF23 in EC cells, we used shRNA and Lipo8000™ transplant reagent (Beyotime, China) targeting KIF23 to transplant Ishikawa and SNGM cells. Cells were harvested 48 h after transfection. RNA interference lentivirus purchased from Beijing Combined Biotechnology Co., Ltd. The target gene sequence for shRNA interference is: sh1 “GCCCAATCCAAAGACTTCT”;sh2 “CCTGTAGACAAGGCAATAT”;sh3 “CCAGCTGGGACCTGGATAT”.

### qRT-PCR

Total RNA from normal and transfected cells was obtained with the RNA Easy Animal RNA Isolation Kit with Spin Column (Beyotime, China). The RNA is then reverse-transcribed into cDNA according to the kit's directions. Then we performed fluorescent quantitative PCR analysis and calculation of the relative expression of the resulting gene based on the 2^(-ΔΔCt) formula. Primers used for experiments: KIF23 (forward primer:5'-CATGGCAAAAATGGTTTTCAAATCT-3' and reverse primers:5'-TGGATTGGGCATAGCTTCTCT-3') and β-actin (forward primer:5′- CTGAGAGGGAAATCGTGCGT-3′ and reverse primers:5′- CCACAGGATTCCATACCCAAG-3′).

### Western blot

We used Ishikawa and SNGM cells during the logarithmic growth period to extract total proteins, and protein content determined by BCA Protein Quantification Kit. Take a protein sample and heat it at 100 °C for 3 min to make the protein fully denatured. After separating the mixed protein sample by using polypropylene gel (10% separation gel, 5% concentrated gel), it was placed onto a polyvinylidene difluoride (PVDF) membrane and sealed with 5% skim milk powder (2.5 g non-fat powdered milk + 50 ml Tris-buffered saline with Tween 20). Incubate these membranes with antibodies at 4 °C for 12–16 h: KIF23 (1:2000; Affinity), p-ERK (1:1000; Beyotime), ERK (1:1000; Beyotime), p-AKT (1:1000; ABclonal), AKT (1:5000; ABclonal), p-PI3K (1:1000; ABclonal), PI3K (1:2000; Bioworld), BCL-2 (1:1000; Beyotime), BAX (1:1000; Beyotime), Caspase-3 (1:1000; Abmart), and GAPDH (1:1000; Beyotime). Subsequently, the sample was coupled with peroxidase-coupled anti-rabbit IgG antibody (1:5000; Beyotime, China) for 2 h under room temperature. ECL enhanced chemiluminescence kit (BOSTER, USA) Using a supersensitive ECL chemiluminescence ready-to-use substrate (BOSTER, USA) to visualize the strip on the Bio-RAD machine.

### Immunohistochemistry

As mentioned earlier, we utilized immunohistochemistry staining to assess protein expression levels. Tissues surgically excised from patients with endometrial cancer were processed into paraffin sections for further analysis. First, we dewaxed the tissue slices and used xylene and gradient alcohol. Then, we performed antigen repair using a sodium citrate antigen repair solution. Next, we placed the tissue slices at room temperature and immersed them in hydrogen peroxide solution for 10 min. Then, we sealed them with sheep serum for another 10 min. We incubated the KIF23 primary antibody (diluted 1:200) in the fridge at 4 °C for 16 h. After 16 h, we removed the slices and rinsed them with PBS. Thirty minutes after rewarming, we added the secondary antibody to the tissue sample and incubated it at room temperature for 12 min. We stained the sections with the DAB Coloring Kit, washed them with tap water, counterstained them with hematoxylin, performed ethanol gradient dehydration, sealed them with xylene and neutral resin, and photographed them under a microscope. Two pathologists independently evaluated all samples to ensure accurate results. We used the H-SCORE to evaluate all samples based on color intensity: 0 for no color, 1 for weakly positive (light yellow or light brown), 2 for positive (brown), and 3 for strongly positive (brown).The Ethics Committee of the First Affiliated Hospital of Jinzhou Medical University has approved this research protocol.

### CCK-8 assay

A control group consisted of Ishikawa and SNGM cells of endometrial carcinoma, while the experimental group consisted of Ishikawa and SNGM cells of endometrial carcinoma with down-expression of KIF23. During the logarithmic growth period, we digested each group of cells with trypsin and resuspended them in complete medium for later use. Inject Ishikawa and SNGM cells into 96-well plates to ensure their cell density is 3 × 103 cells/well. Every 24 h, we should remove the 96-well plates from the incubator. Add 10 ul CCK-8 solution per well, paying attention to the deflective operation. Finally, we put the 96-hole plate in the incubator for about 90 min, measuring the absorption value at 450 nm.

### Wound healing assay

The control group, consisting of Ishikawa and SNGM cells, and the experimental group, consisting of Ishikawa and SNGM cells with down-expression of KIF23, were digested by trypsin and then suspended into cell suspension by complete culture. Then, spread (5–10) × 105 cells on a six-well plate and let it grow full overnight. The next day, lines perpendicular to the reverse side of the six-well plate were drawed with a pipette tip, then wash those floating cells with PBS. Add serum-free medium to the 6-well plates, Subsequently photographed under a microscope as a result of a 0 h experiment. The same field of 0 h vision is imaged again after 24 h. The healing area was finally measured using Image J software.

### Colony formation

Again, the control group consisted of Ishikawa and SNGM cells, while the experimental group consisted of Ishikawa and SNGM cells with down-expression of KIF23. We digested each group of cells during the logarithmic growth period with trypsin and resuspended them in a complete culture. Each cell suspension was diluted to 1000 cells per well, inoculated into a six-well plate, gently shaken to disperse them evenly, and cultivate the cells in the incubator for two weeks. Ensure that the incubator temperature is 37 °C, and keep the CO2 concentration adjusted at 5%. When we find visible clones of cells in the 6-well plates, we can stop the cell culture. After removing the liquid, wash with PBS two times. Fix the cells with 100% methanol for 15 min, then remove the methanol. Subsequently, add a moderate amount of crystal violet dye for 10 min. The dyeing solution was washed with PBS and dried at room temperature. Finally, the microscope collects images, and the Image J software performs image analysis.

### Transwell migration assay

For the transwell experiment, Ishiwawa-nc, Ishikawa-shKIF23, and SNGM-nc and SNGM-shKIF23 adjusted cell density to 2.0 × 104/well and 3.0 × 104/well. In the upper chamber, the suspension of each group of cells with 200 μl of non-serum medium suspended, while the lower chamber received 800 μl of medium containing 20% serum. After 12–48 h of cell incubation, fix the cells 30 min with 100% methanol and add crystal violet for 10 min. The microscope collects images, and the ImageJ software performs image analysis.

### Animal experiment

The animals used in this experiment were SPF grade Balb/c female mice. We acquired 3 ~ 4-week-old female nude mice(Balb/c) from Liaoning Changsheng Biotechnology Co., Ltd. for using in the experiment. We injected the Ishikawa cells transplanted with KIF23-shRNA and the controlled group Ishikaw cells into two groups of mice(Balb/c), then measured the volume of tumor every three days. The results indicated a significant decrease in tumor volume within the KIF23-shRNA group. After the two sets of specimens were fixed and embedded in paraffin, we underwent an immunohistochemical assay to detect the expression of KIF23. The studies have revealed that the KIF23 knock-down group exhibited reduced expression of KIF23 in comparison to the control group. The formula for calculating tumor volume is: volume = width^2 × length × 0.5. The Ethics Committee of the Jinzhou Medical University approved the animal research experiments(2,022,120,801).

### Statistical analysis

Data analysis was conducted using ANOVA or t-test, and the data is presented as the mean ± standard deviation in GraphPad Prism 9.

## Results

### The expression and prognosis of KIF23 in endometrial cancer

**Fig. 1:**
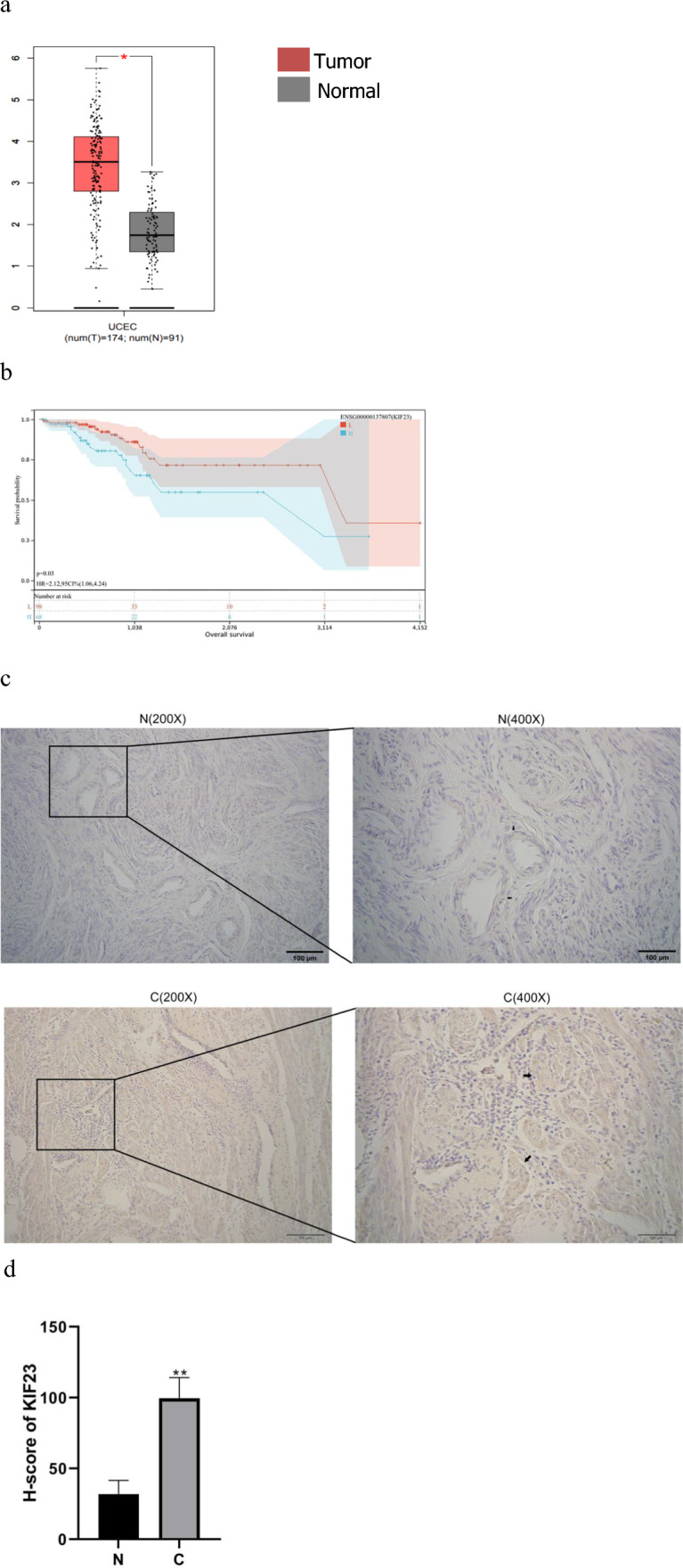
The gene KIF23 expression and prognosis in endometrial cancer. **a** KIF23 expression is markedly higher in 174 tumor tissues compared to 91 cancer-adjacent tissues (P < 0.05). **b** Overexpression KIF23 in endometrial cancer patients exhibited poor overall survival (OS). **c** The cytoplasm and nucleus was the predominant location of positive staining for KIF23. Weak staining observed in normal endometrial tissue (N). and strong staining observed in endometrial cancer tissue (C). **d** The H-SCORE for the cancer tissue (C) and the adjacent tissues (N) (P < 0.01)

To investigate the relationship between KIF23 and the development of endometrial cancer, we analyzed KIF23 mRNA expression using the GEPIA database and corresponding endometrial cancer dataset. The analysis revealed higher expression levels of KIF23 in EC tissues compared to adjacent tissues (Fig. 1a). Furthermore, increased KIF23 expression in endometrial cancer patients is associated with a poor prognosis (Fig. 1b). Based on these findings, KIF23 may play a role in the poor prognosis of endometrial cancer patients. To confirm these findings, we evaluated the expression levels of KIF23 protein in endometrial carcinoma tissues and normal endometrium obtained from the First Affiliated Hospital of Jinzhou Medical University. Immunohistochemical analysis showed that tumor tissues of endometrial cancer expressed higher levels of KIF23 compared to normal endometrium (Fig. 1c). The H-SCORE for the cancer tissue was 99.65 ± 11.97, while the H-score for the adjacent tissues was 31.99 ± 7.81, representing a statistically significant difference (P < 0.01) (Fig. 1d).

### The expression of KIF23 in Ishikawa and SNGM was reduced by shRNA targeting KIF23

**Fig. 2:**
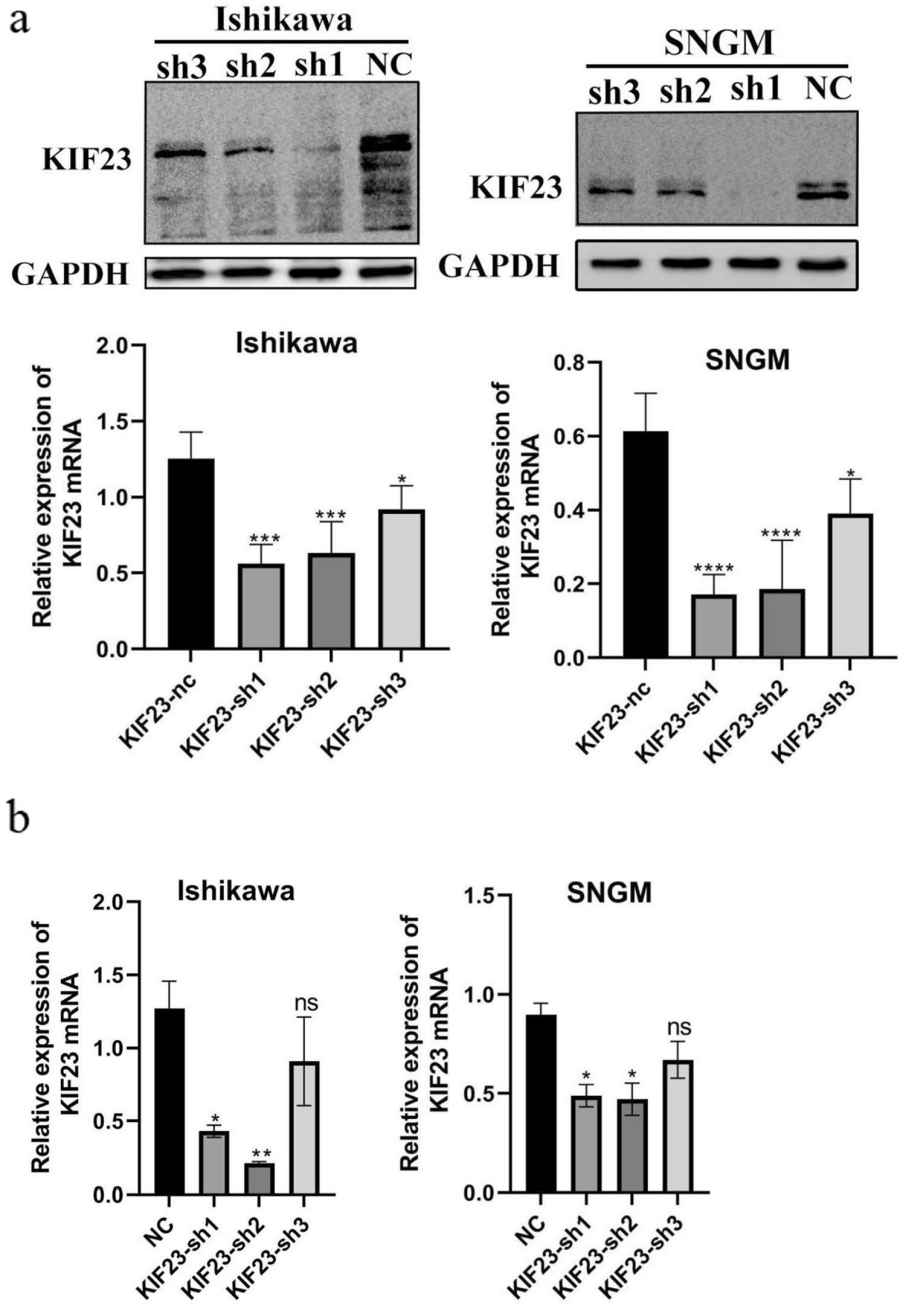
The knocking down efficiency of KIF23. a after transfection, Western blot analysis revealed low levels of KIF23 expression. b PCR analysis showed low KIF23 expression when Ishikawa and SNGM cells were transfected with shRNA. *P < 0.05,**P < 0.01, ***P < 0.001. ns, no significance

The aim of our study was to investigate the role of KIF23 in tumor growth. To achieve this, we used shRNA to silence KIF23 expression in Ishikawa and SNGM cells. We confirmed the efficiency of KIF23 knockdown using qRT-PCR and Western blotting techniques. The studies demonstrated that KIF23-sh1 and KIF23-sh2 had higher knockdown efficiency (Fig. 2a,b).

### KIF23 knockdown inhibits endometrial cancer cell proliferation, colony formation and migration

**Fig. 3:**
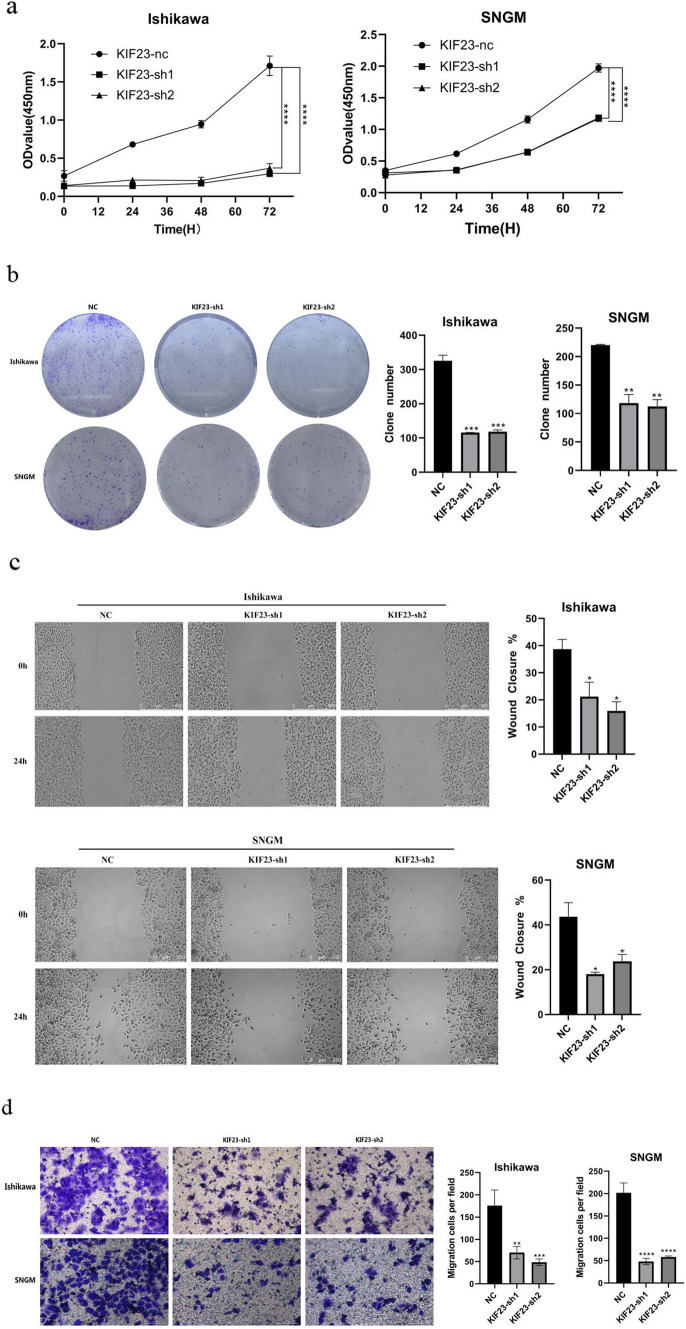

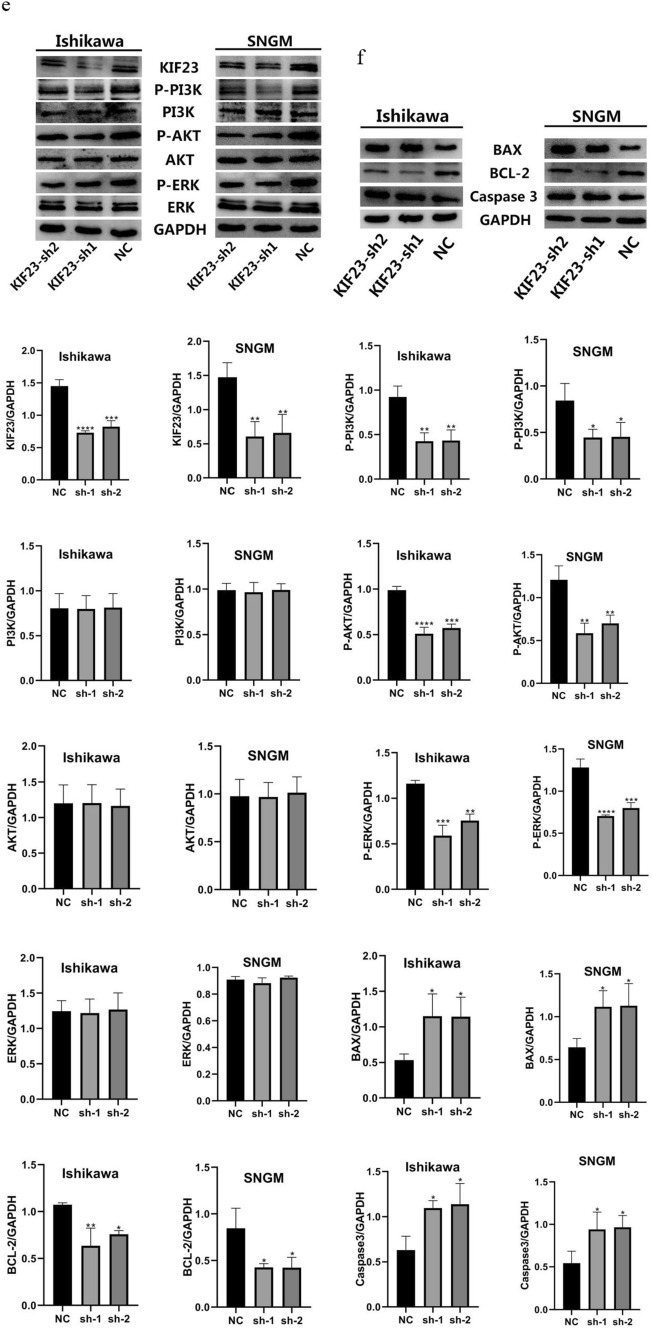
Knockout KIF23 interferes with growth in Ishikawa and SNGM cell lines. **a**, **b** The outcomes of the CCK8 and colony formation experiments revealed shKIF23 effects on the proliferation of Ishikawa and SNGM cells (**c**, **d**) KIF23 knockout during wound healing experiments and transwell analysis inhibited migration of Ishikawa and SNGM cells. **e** KIF23 was knocked down in Ishikawa and SNGM cells, Western blotting results proved that KIF23 knocked down-regulated P-PI3K, p-ERK, and p-AKT expression. **f** KIF23 knockdown reduced BCL-2 expression related to apoptosis and increased the expression of BAX, caspase-3 proteins. *P < 0.05,**P < 0.01, ***P < 0.001,****P < 0.0001

We transfected Ishikawa and SNGM cells with KIF23-sh1 and KIF23-sh2, respectively. Subsequently, we observed changes in cell proliferation, colony formation, and migration. CCK-8 analysis demonstrated that KIF23 knockdown significantly inhibited endometrial cancer cell proliferation (Fig. 3a). Similarly, colony formation assays revealed that downregulating KIF23 decreased colony formation in Ishikawa and SNGM cells (Fig. 3b). Furthermore, we evaluated endometrial cancer cells' migration ability using wound healing and Transwell migration assays. The results demonstrated that knocking down KIF23 hindered Ishikawa and SNGM cell migration (Fig. 3c, d). These findings suggest that KIF23 knockout may inhibit endometrial cancer cell proliferation, colony formation, and migration.

### The role of KIF23 in endometrial cancer

By testing the activation levels of signal transduction pathways in KIF23-transplanted and knockdown cells, we observed that reducing KIF23 expression decreased ERK and AKT/PI3K pathway activation (Fig. 3e). Additionally, Western blot analysis revealed that KIF23 knockdown promotes apoptosis, leading to reduced BCL-2 expression and increased levels of BAX and caspase-3 proteins (Fig. 3f). Collectively, these results support the conclusion that KIF23 promotes EC cell proliferation and inhibits apoptosis by activating ERK and AKT/PI3K signaling pathways.

### Knockout of KIF23 inhibits tumor growth in mice

**Fig. 4:**
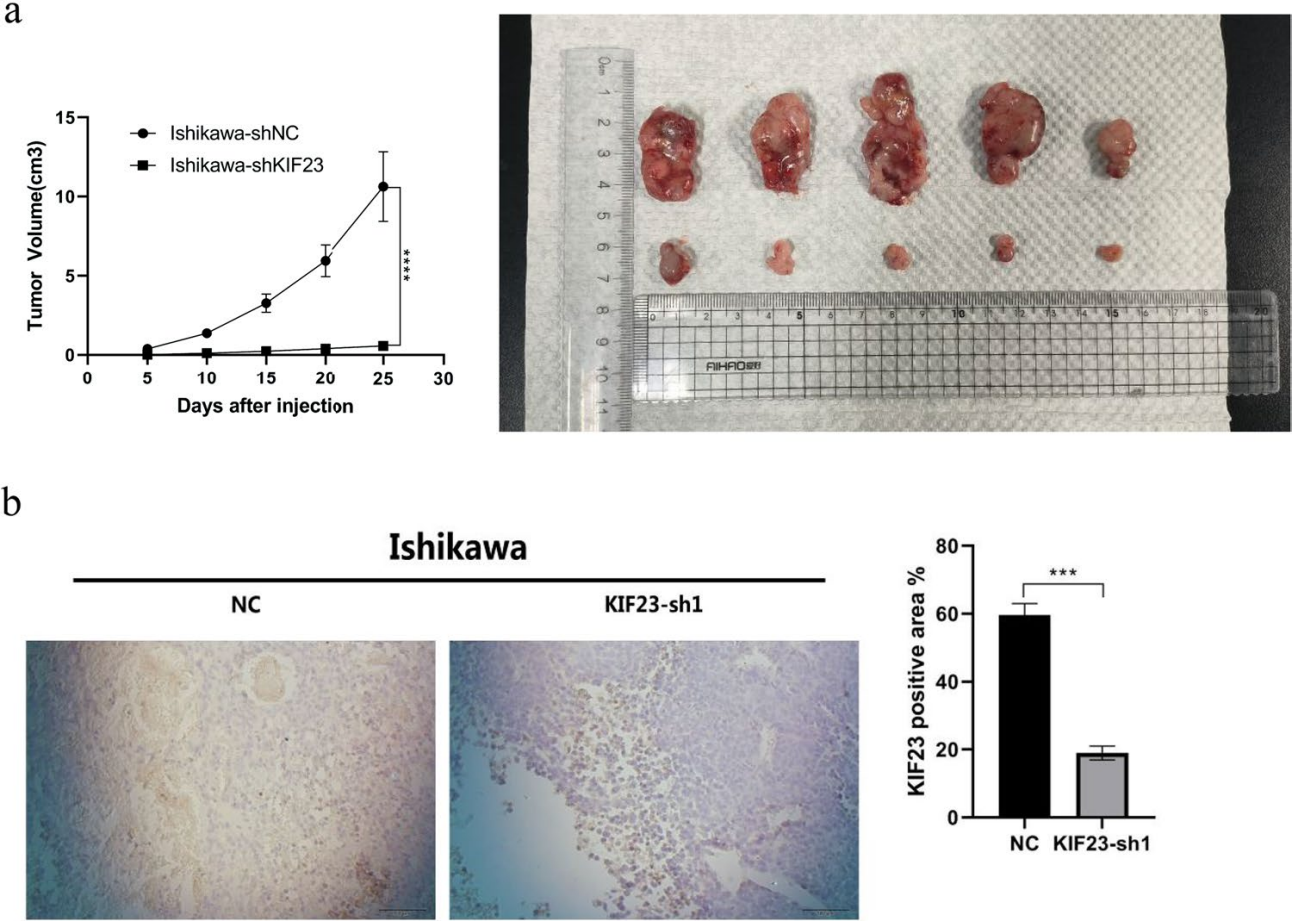
A mouse xenotransplantation model was used to analyze KIF23 function in vivo. **a** The tumor growth curve of Ishikawa cells transfected with KIF23-shRNA was obviously inhibited. **b** Immunohistochemistry revealed a decrease in KIF23 expression in the knockdown KIF23 group in comparison to the control group. ***P < 0.001,****P < 0.0001. Scale = 100 μm

To evaluate the role of KIF23 in mice, we injected Ishikawa cells transfected with KIF23-shRNA and control Ishikawa cells into nude mice (Balb/c). Subsequently, tumor size was measured in both groups. Notably, the shKIF23 group exhibited significant tumor growth inhibition (Fig. 4a). Furthermore, immunohistochemical staining confirmed that KIF23 expression was reduced in the knockout group compared to the control group (Fig. 4b), providing further evidence for effective KIF23 knockdown in the shKIF23 group. Consequently, these findings indicate that KIF23 plays a crucial role in cancer initiation and progression, offering novel insights and potential therapeutic options for cancer treatment.

## Discussion

Endometrial cancer, the most prevalent malignancy in the female reproductive tract, has seen a consistent rise in both incidence and mortality rates [29, 30]. Currently, the standard treatment options for endometrial cancer include surgery, radiotherapy, and chemotherapy [31–33]. Early-stage endometrial cancer is often curable; however, progress in treating advanced and recurrent cases has been limited [34–36]. Despite ongoing efforts, the prognosis for endometrial cancer remains unsatisfactory, emphasizing the urgent need to investigate innovative treatment strategies to improve outcomes. Therefore, a deeper understanding of the underlying mechanisms of endometrial cancer is crucial [37–40]. Our research has demonstrated a correlation between elevated KIF23 levels and poor patient outcomes. Additionally, low KIF23 expression is associated with longer survival durations [41]. We propose that KIF23 may serve as a potential prognostic marker for endometrial cancer.

Our research has shown that specifically targeting KIF23 with shRNA can effectively inhibit endometrial cancer cell growth both in vitro and in vivo. Two distinct shRNA sequences were used in our experimental study to target KIF23. Previous studies have also demonstrated KIF23's role in promoting the proliferation of various cancers, including breast cancer [42], bladder cancer [43], and hepatocellular cancer [44]. When compared to control cells, stable clones of KIF23 knock-down cells exhibited slower growth rates in vivo. Notably, KIF23 not only functions as a regulator of cytokinesis but also acts as a microtubule motor enzyme. The significant upregulation of KIF23 in endometrial cancer compared to normal endometrial tissue strongly suggests that KIF23 could be a promising therapeutic target for effective endometrial cancer treatment [45]. In summary, our research indicates that KIF23 may have a critical part in the development of EC. Additionally, it also promotes cancer cell proliferation and impedes apoptosis. However, our study did have some limitations. Specifically, we only utilized patient samples from one center, which may not be representative of other populations. Additionally, our sample size was relatively small. Nonetheless, we did our best to account for these limitations in our analysis and believe that our findings offer valuable insights into the study.

To summarize, our findings suggest that KIF23 plays a crucial role in the development of endometrial cancer. Furthermore, it promotes cancer cell proliferation and inhibits apoptosis. Nevertheless, it should be noted that our study had some limitations. Firstly, the patient samples were obtained from only one center, potentially limiting the generalizability of our findings. Additionally, the sample size was relatively small. Despite these limitations, we took measures to address them in our analysis and believe that our findings provide valuable insights into the study.

### Supplementary Information

**Additional file 1.** Raw data from western blotting.

## Data Availability

The data underlying this article are available in the article and its online supplementary material.
