# Supplementary material for: Mechanism of regulation of KIF23 on endometrial cancer cell growth and apoptosis
Source: Discov Oncol. 2024 Mar 21;15:83. doi: 10.1007/s12672-024-00937-x (PMC10957832; doi:10.1007/s12672-024-00937-x)

Raw data from western blotting to：

Original article：

MECHANISM OF REGULATION OF KIF23 ON ENDOMETRIAL CANCER CELL

GROWTH AND APOPTOSIS

Ruiying Zhuang1,Haiyan Liu2

1 Jinzhou Medical University, Jinzhou, Liaoning Province, China. ORCID:(https://orcid.org/0009-0004-2105-446X)

2 The First Affiliated Hospital of Jinzhou Medical University, Jinzhou, Liaoning Province, China. ORCID:( https://orcid.org/0000-0002-7610-4119)

* corresponding author: Haiyan Liu, The First Affiliated Hospital of Jinzhou Medical University, Jinzhou, Liaoning Province, China.

E-mail: 448546815@qq.com

Tel:+86-180-41657437

Raw data from western blotting

**Note**: Some bands incubate other antibodies after being soaked in strippingbuffer.

KIF23(110KDa)

Figure 2a (Ishikawa)


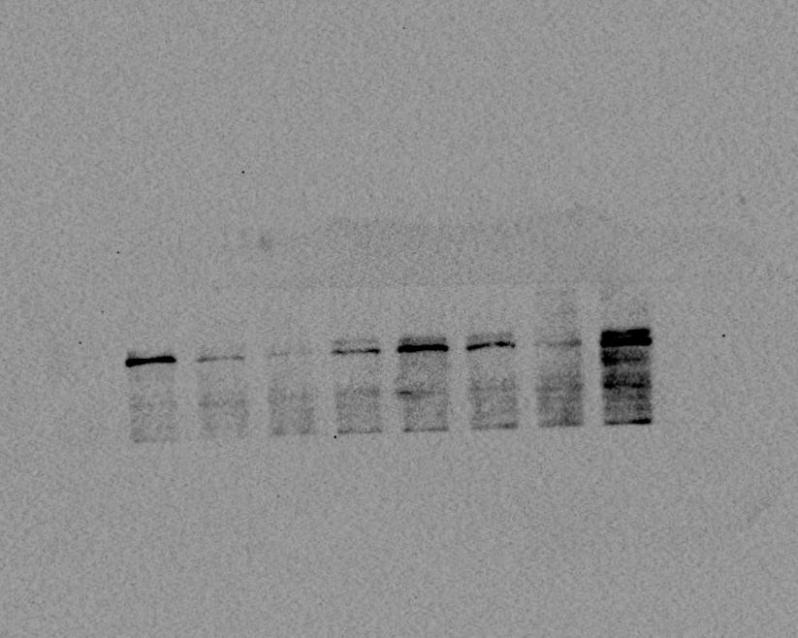

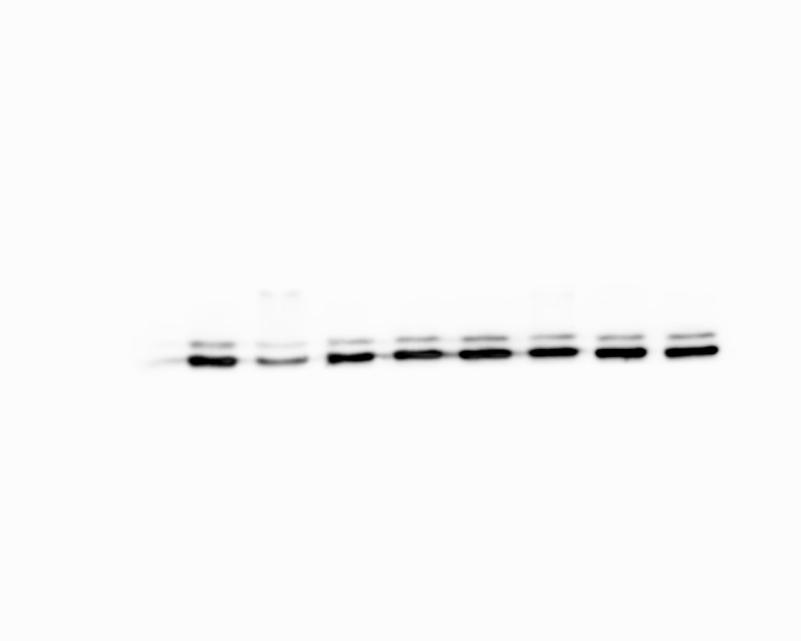


KIF23(110KDa)

Figure 2a (SNGM)


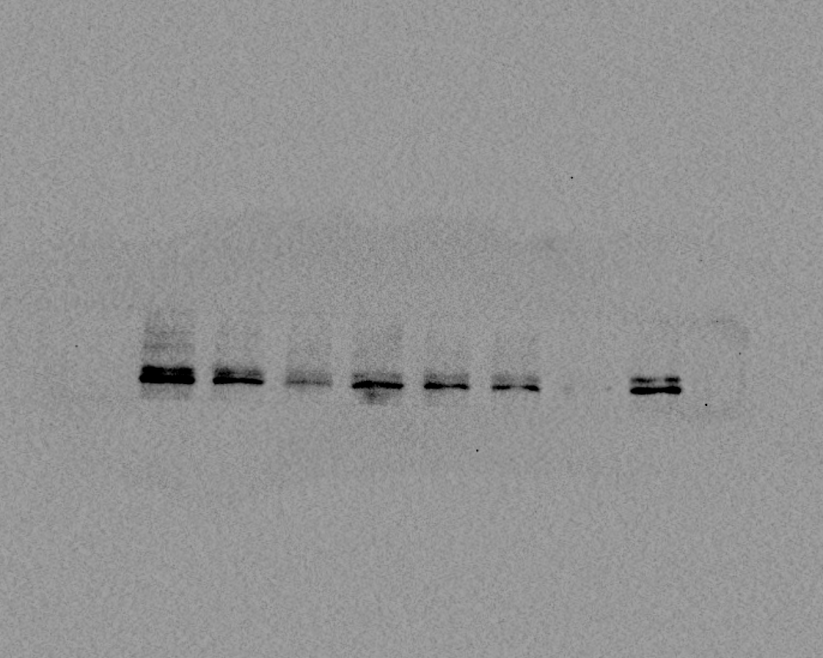


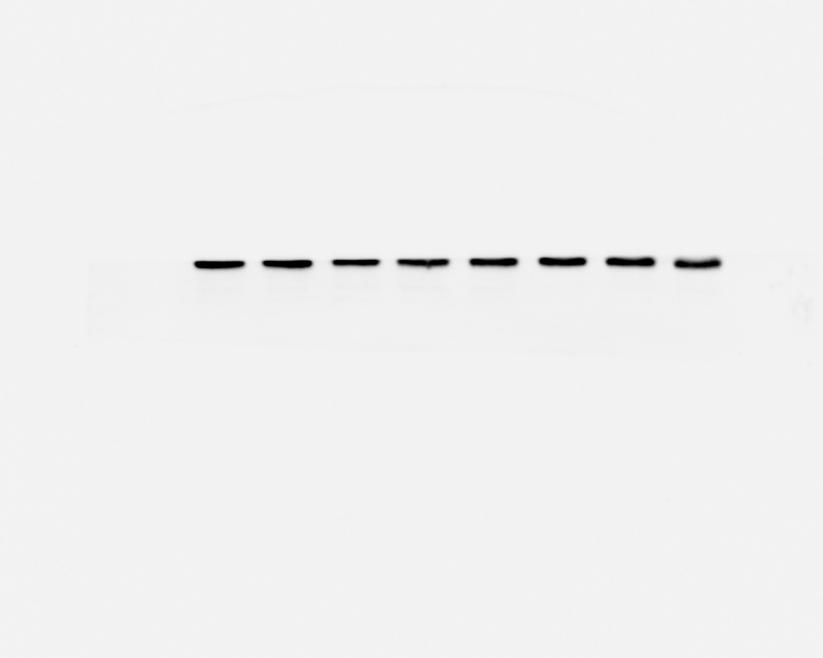


KIF23(110kDa)

Figure 3e(Ishikawa)


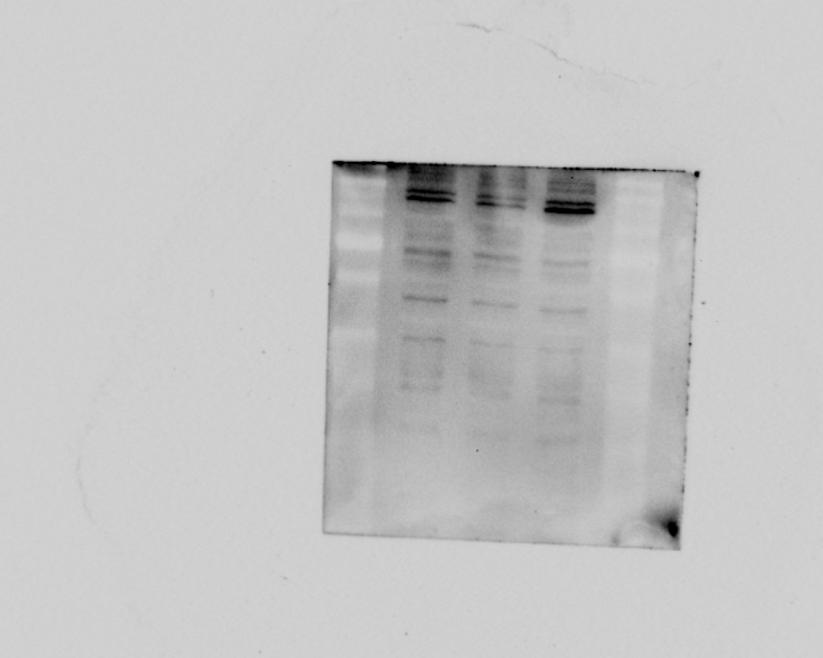


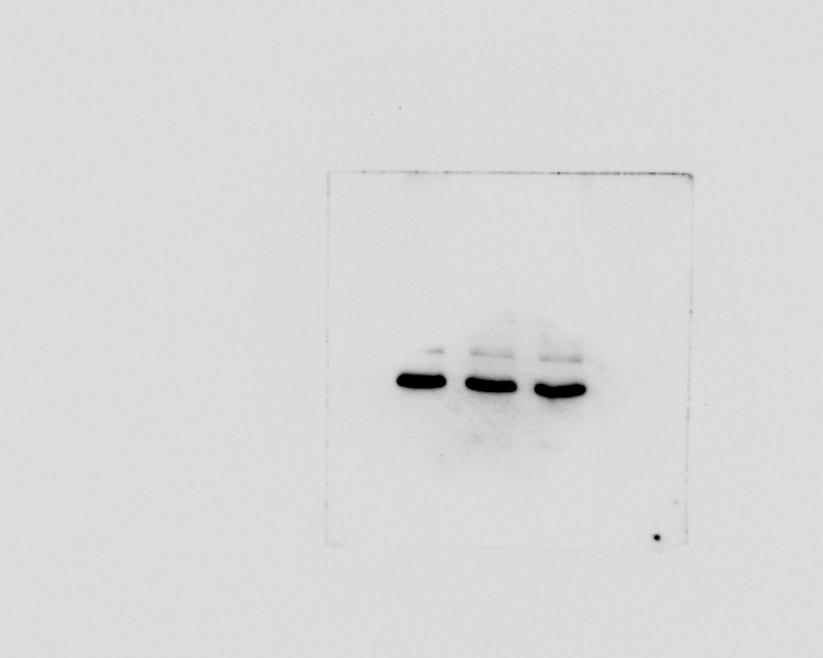


KIF23(110kDa) Figure 3e(SNGM)


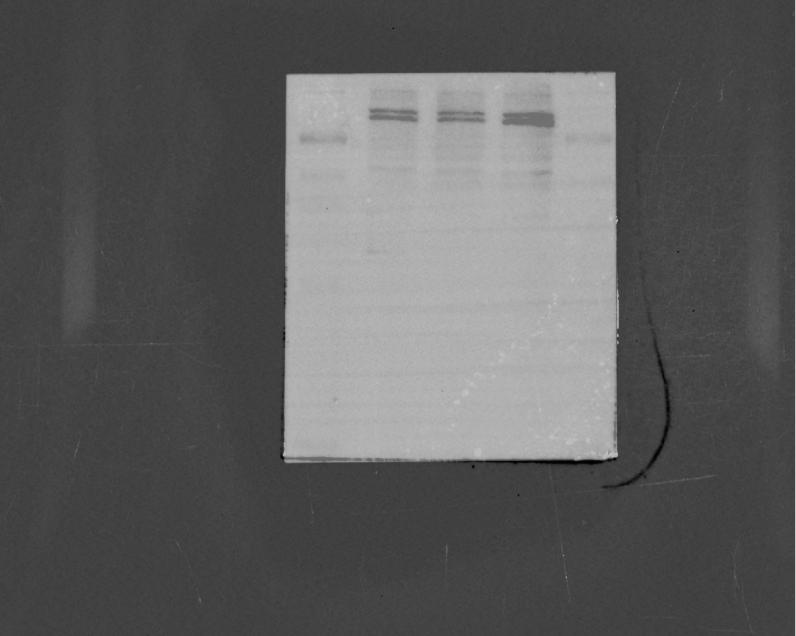


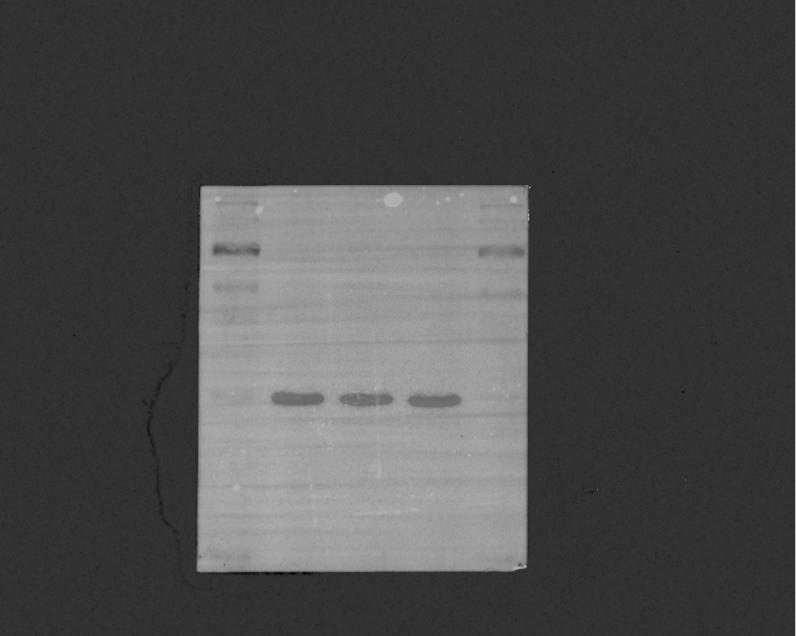


P- PI3K(85KDa)

Figure 3e(Ishikawa)


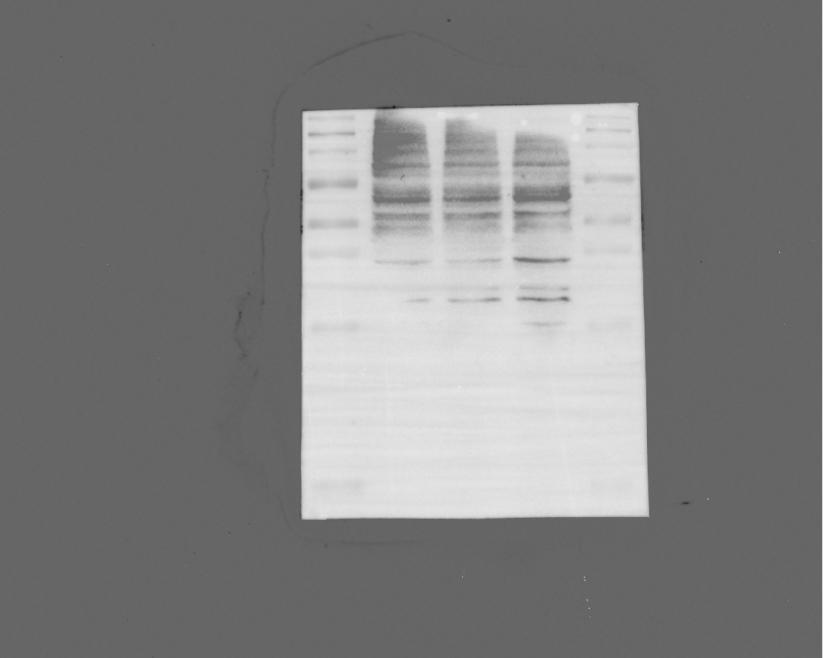


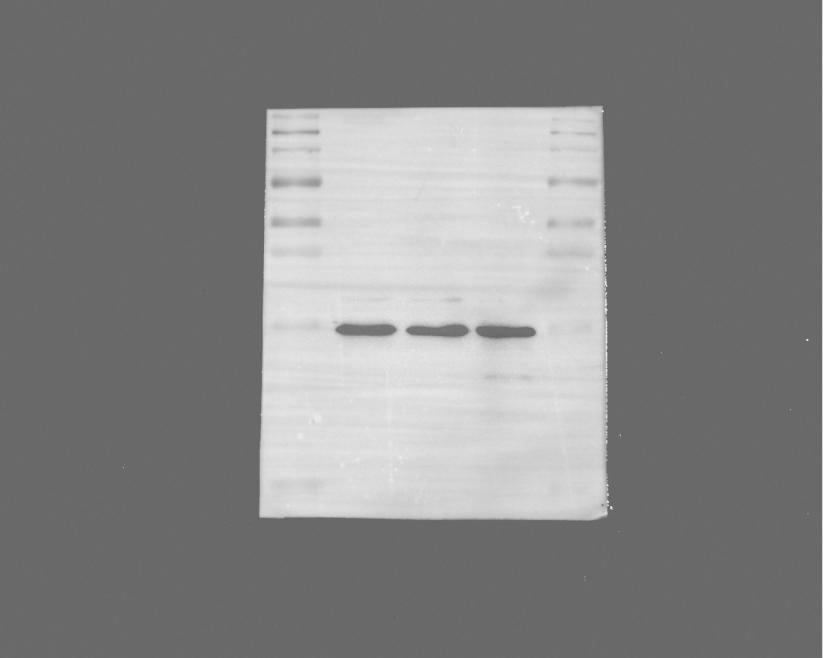


P-PI3K(85KDa)

Figure 3e(SNGM)


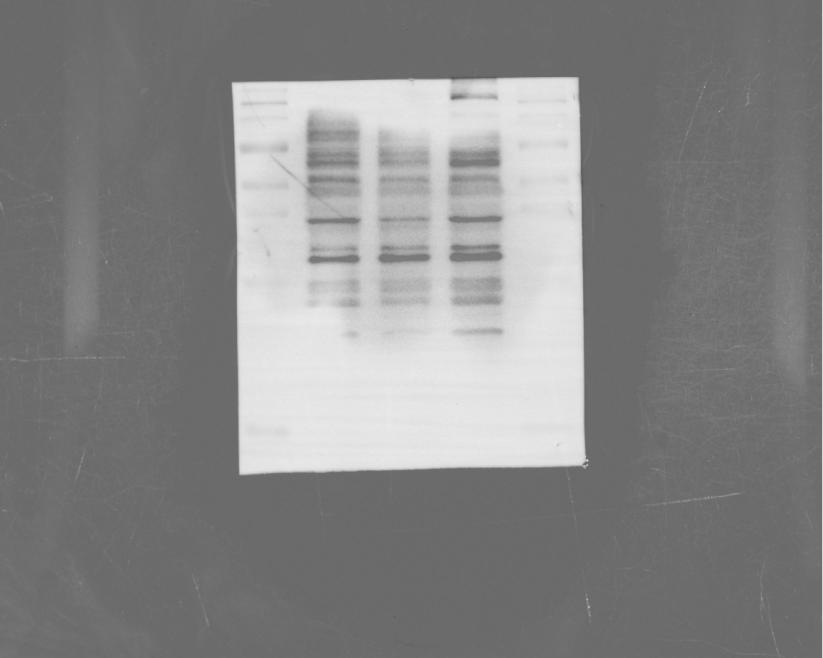


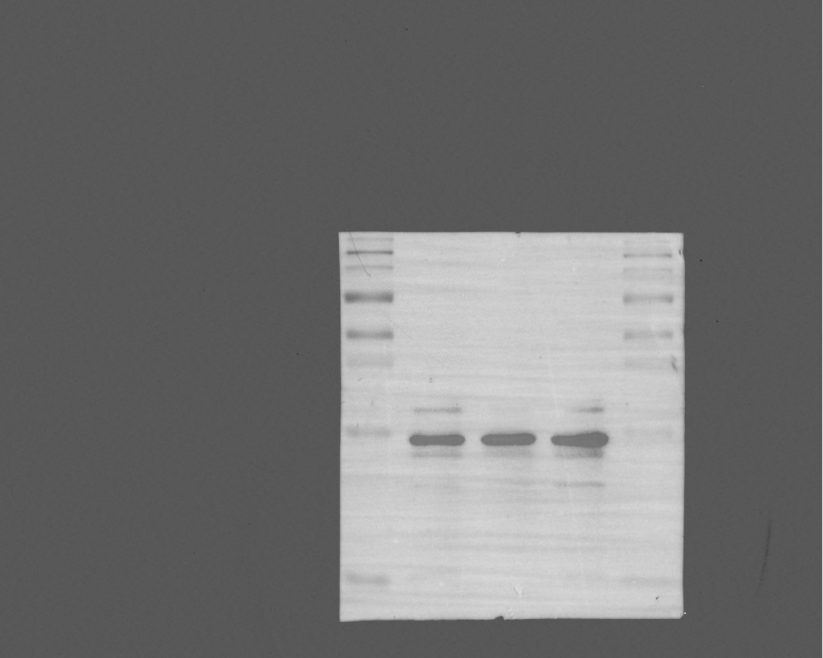


PI3K(85KDa)

Figure 3e(Ishikawa)


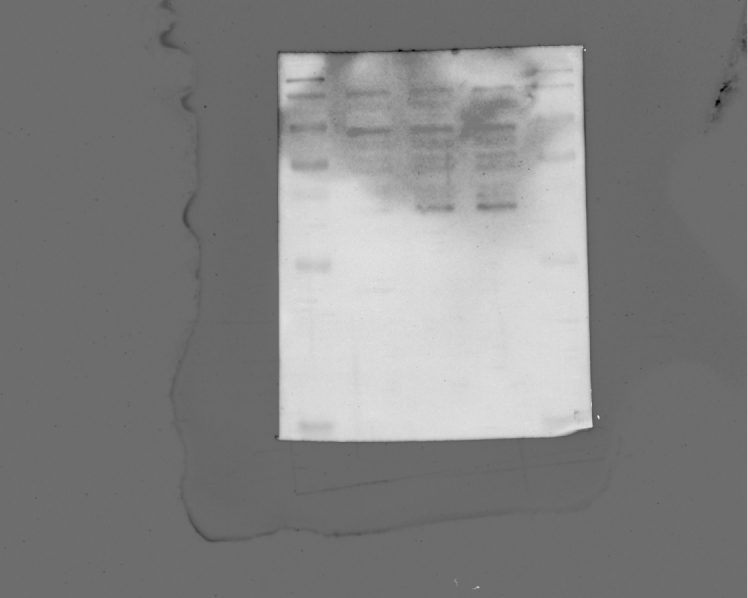


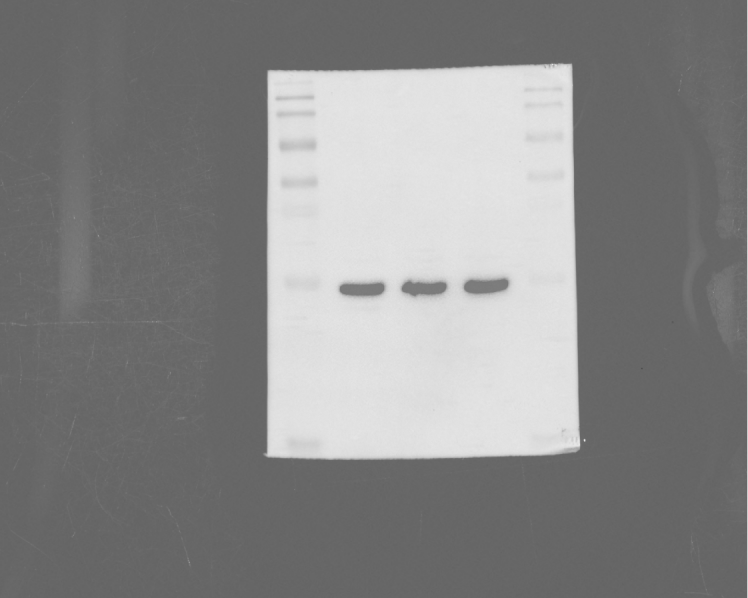


PI3K(85kDa)

Figure 3e(SNGM)


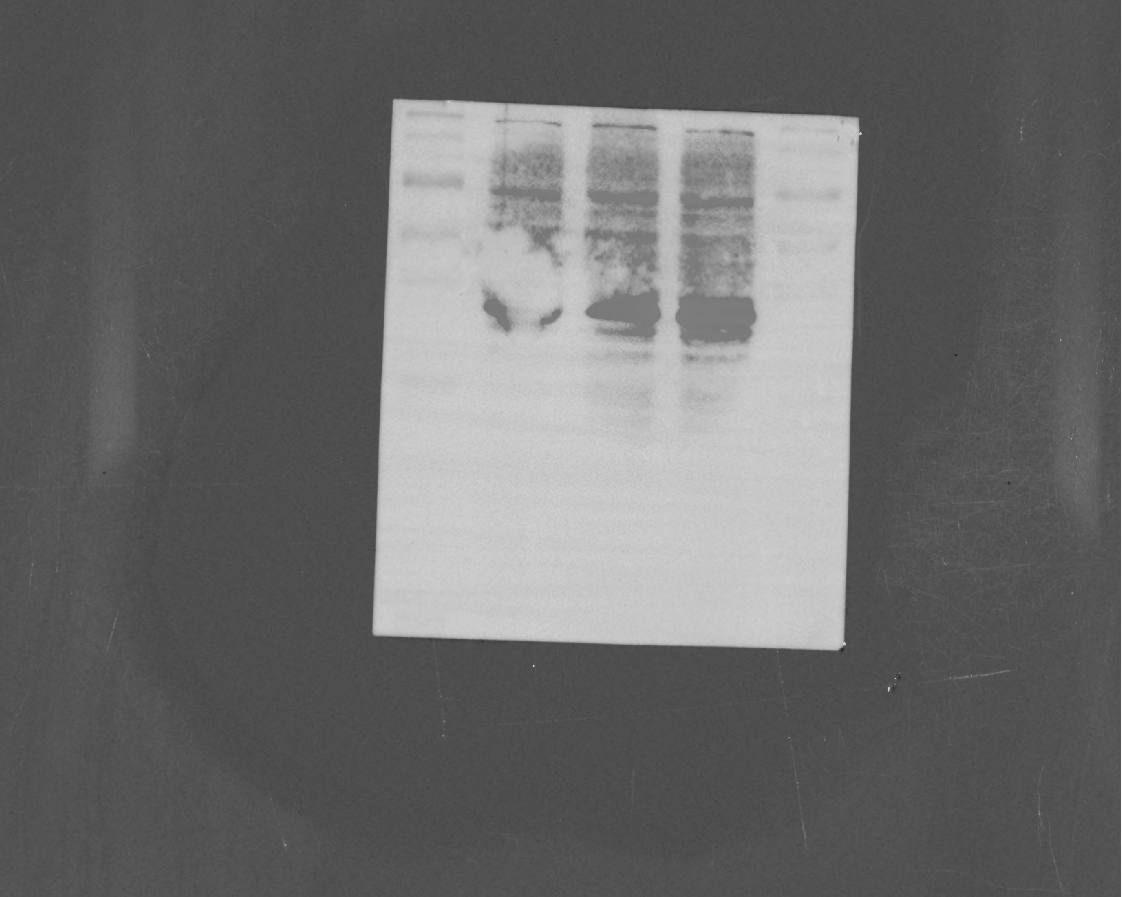


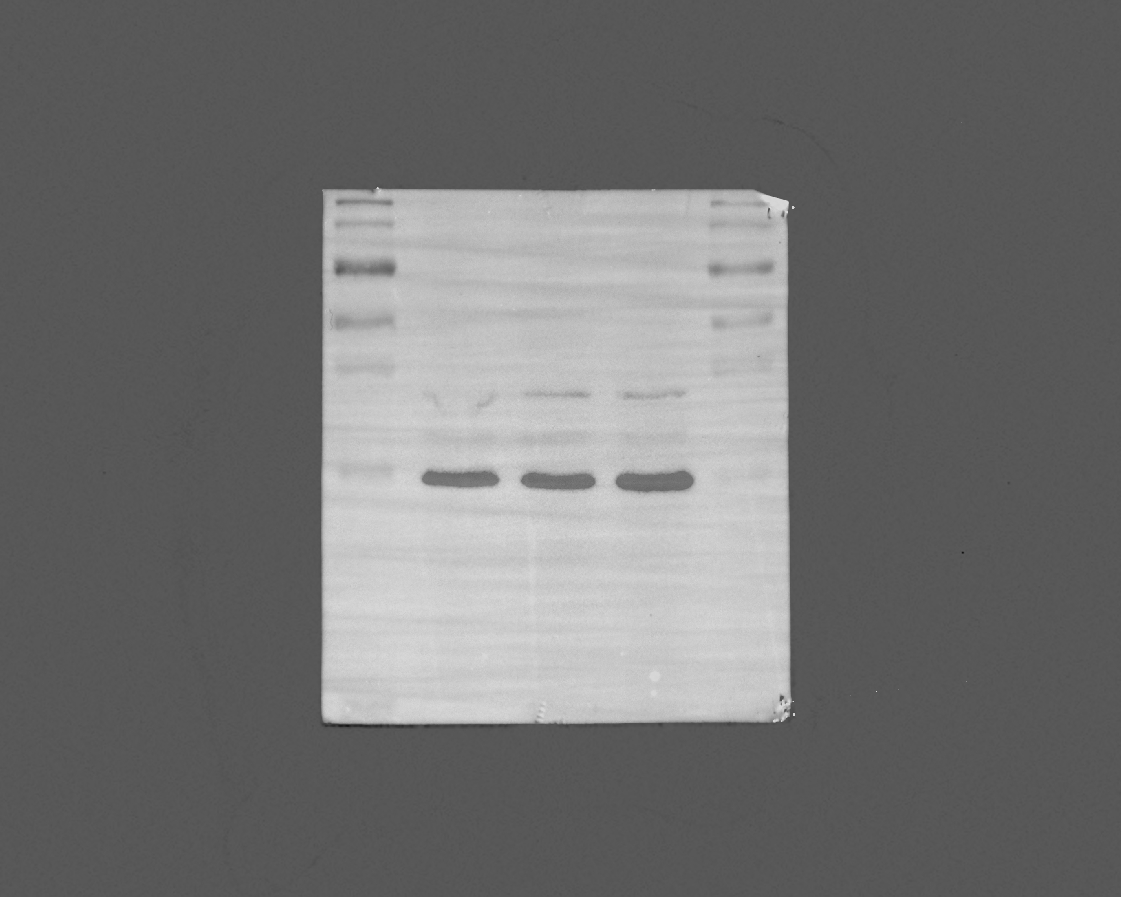


P-AKT(60kDa)Note: The theoretical molecular weight of P-AKT is 60, and the actual experimental result is about 66.

Figure3e(Ishikawa)


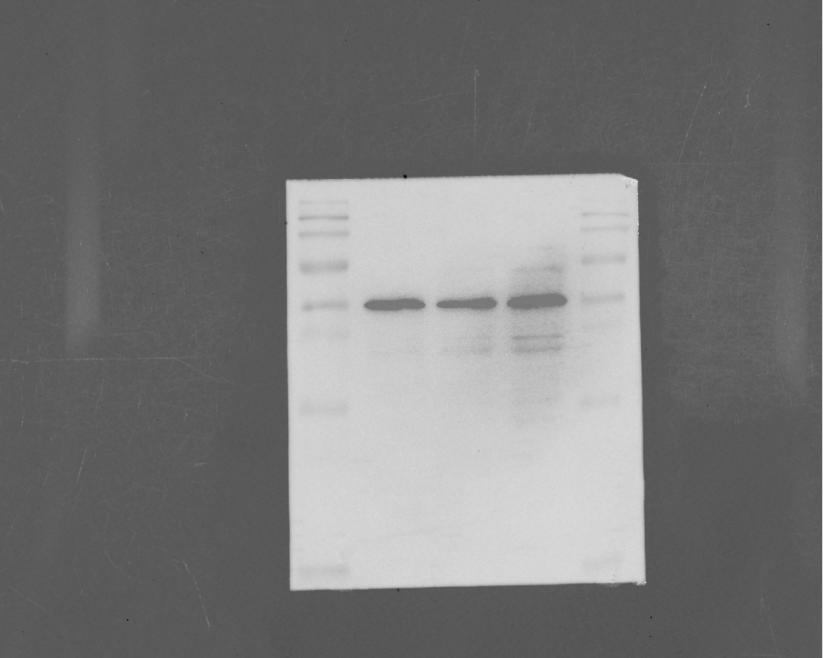


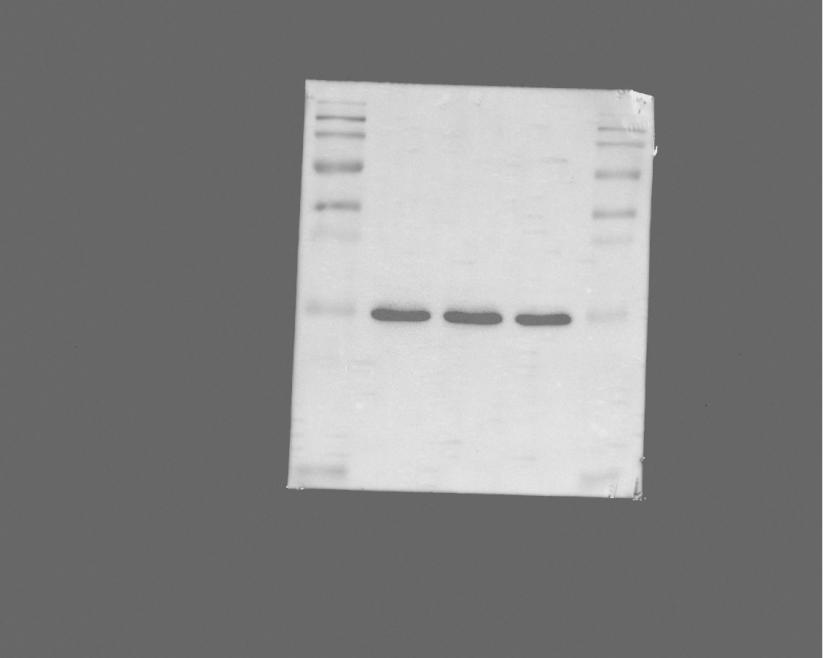


P-AKT(60KDa)

Figure 3e(SNGM)


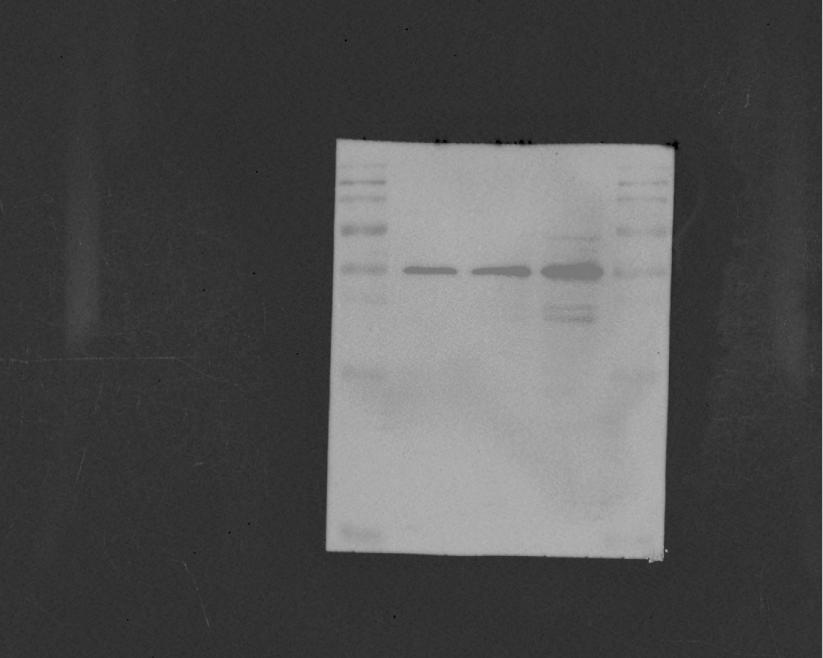


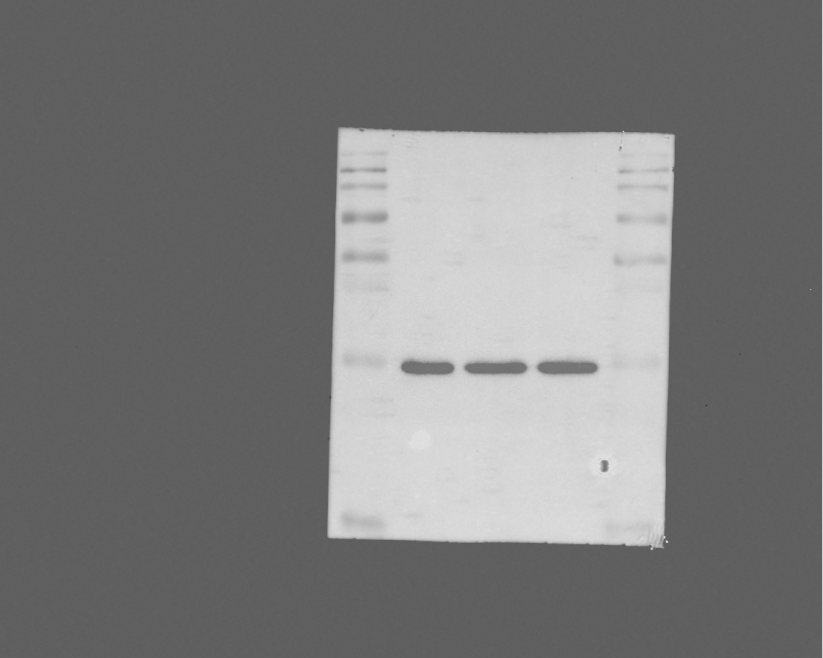


AKT(60kDa)Note: The theoretical molecular weight of P-AKT is 60, and the actual experimental result is about 66.

Figure3e(Ishikawa)


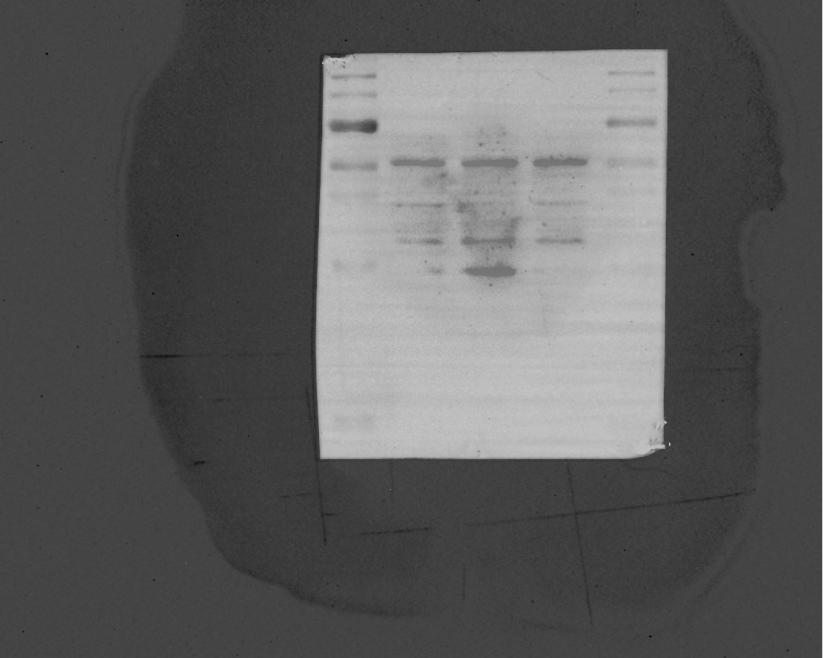


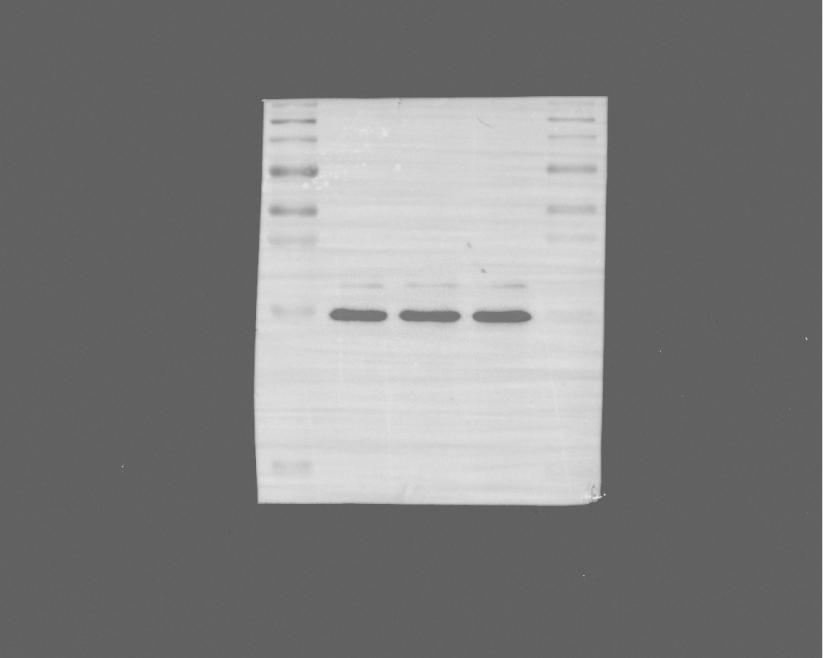


AKT(60KDa)

Figure 3e(SNGM)


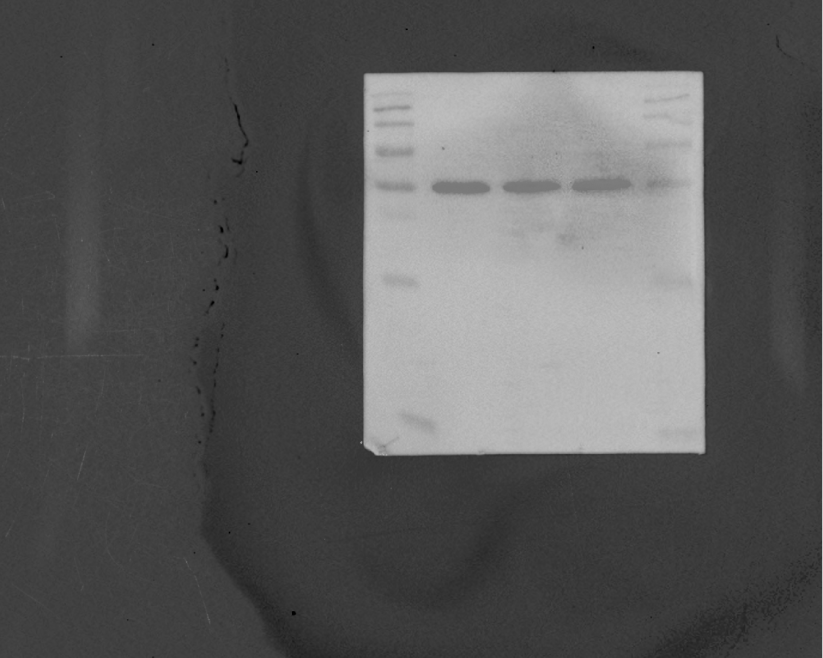


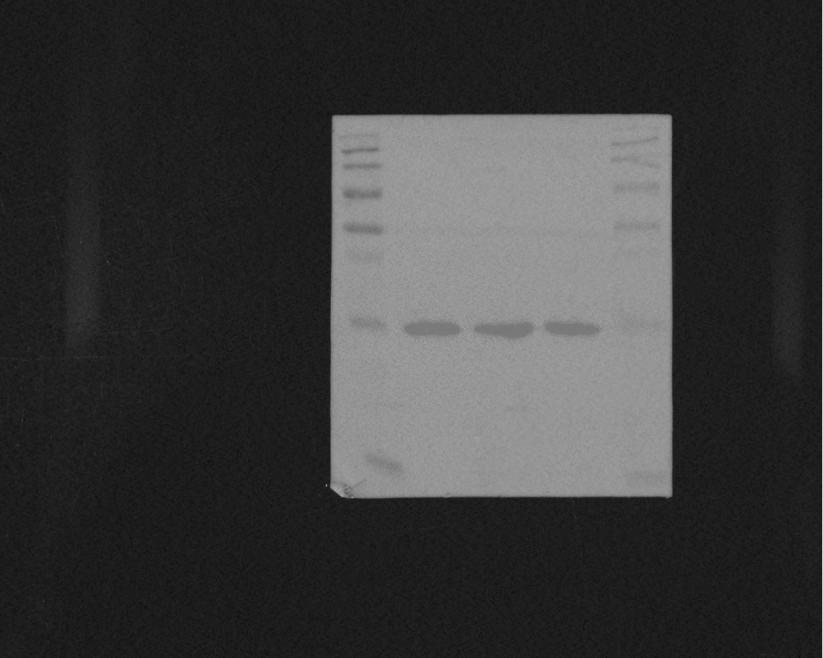


P-ERK(42KDa)

Figure 3e(Ishikawa)


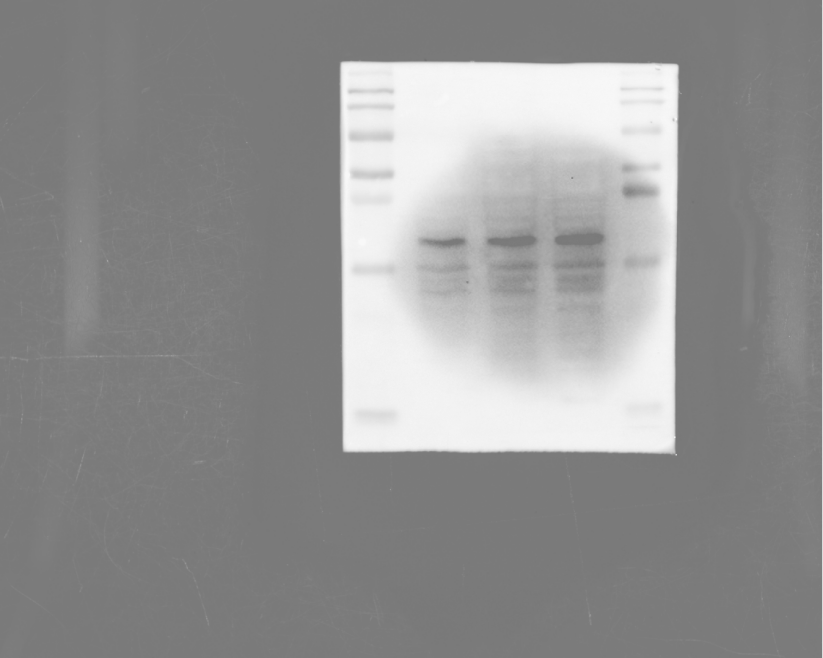


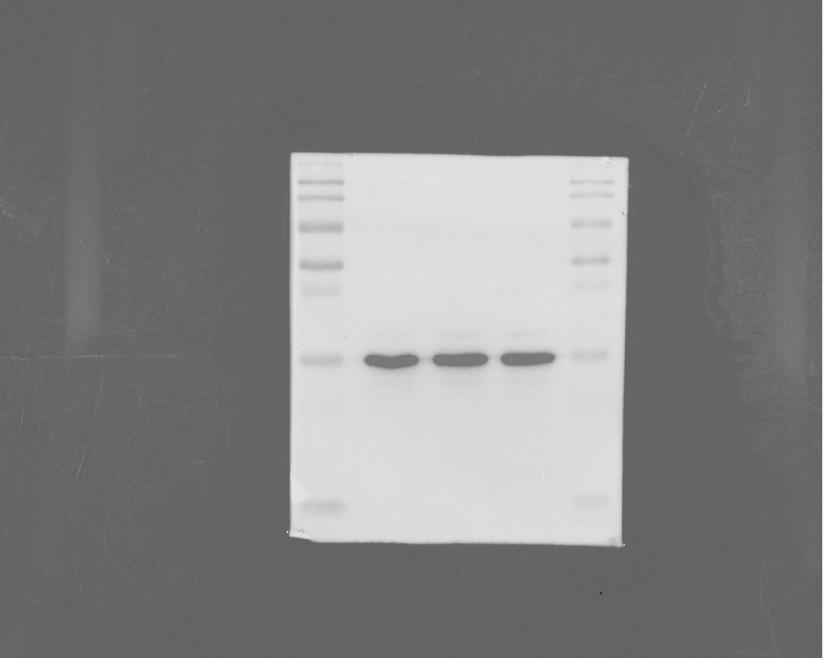


1. ERK(42KDa)

Figure 3e(SNGM)


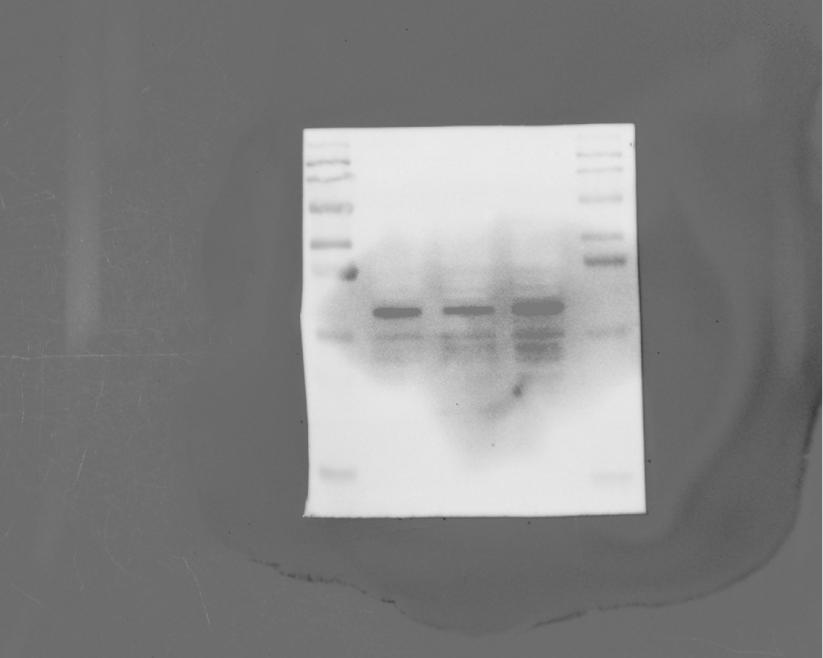


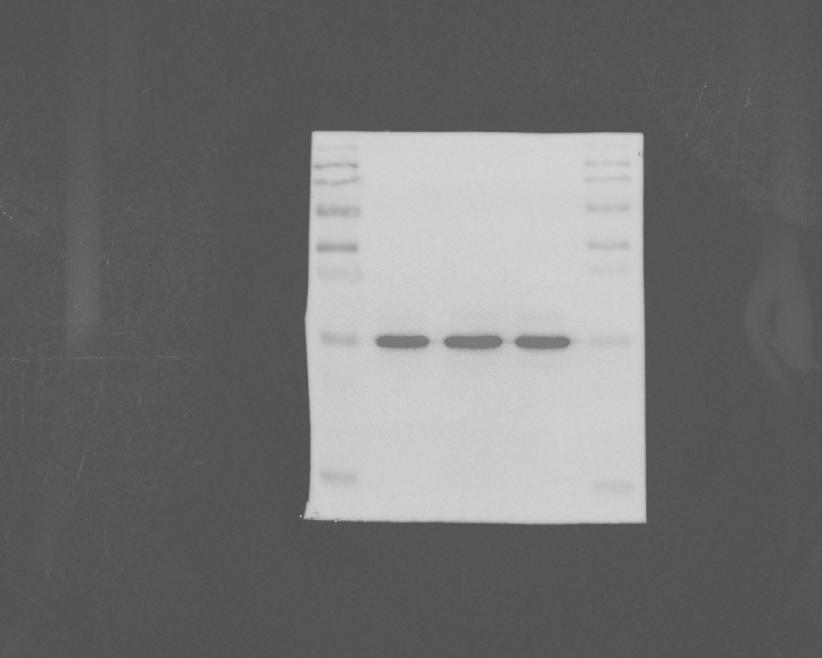


ERK(42KDa)

Figure 3e(Ishikawa)


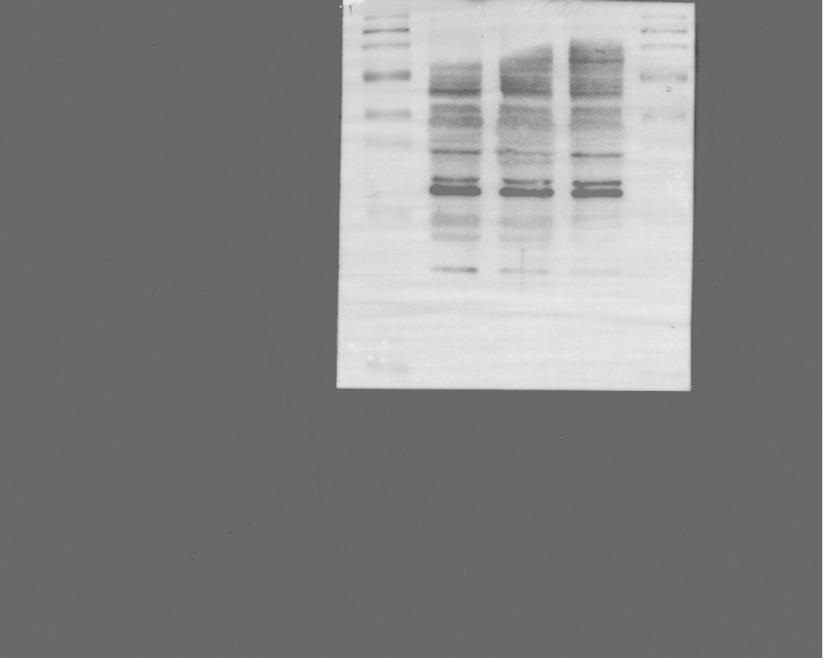


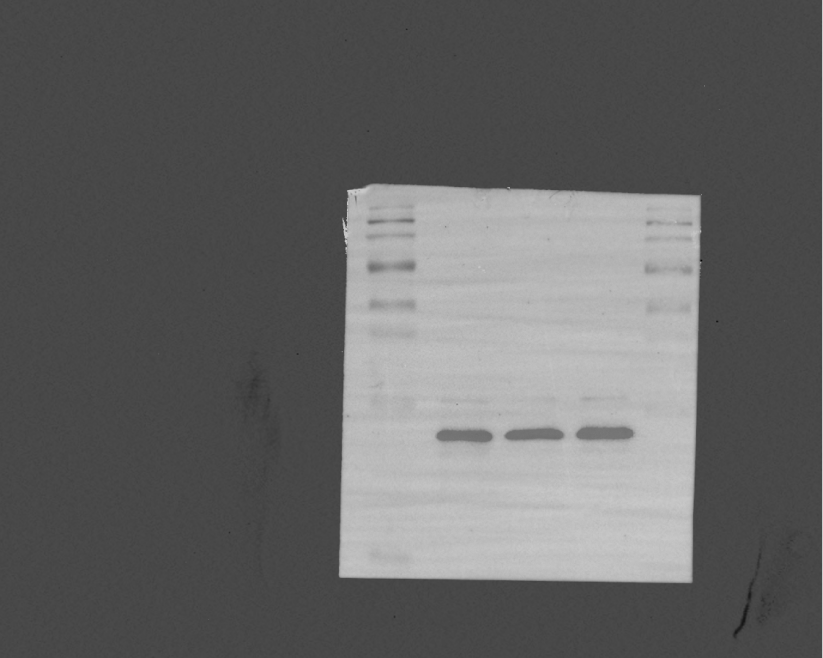


ERK(42KDa)

Figure 3e(SNGM)


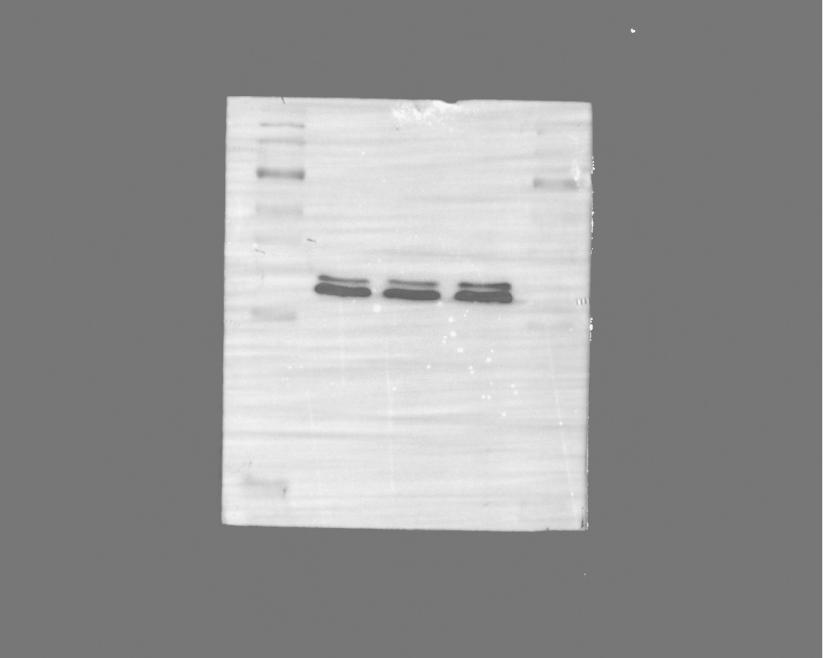


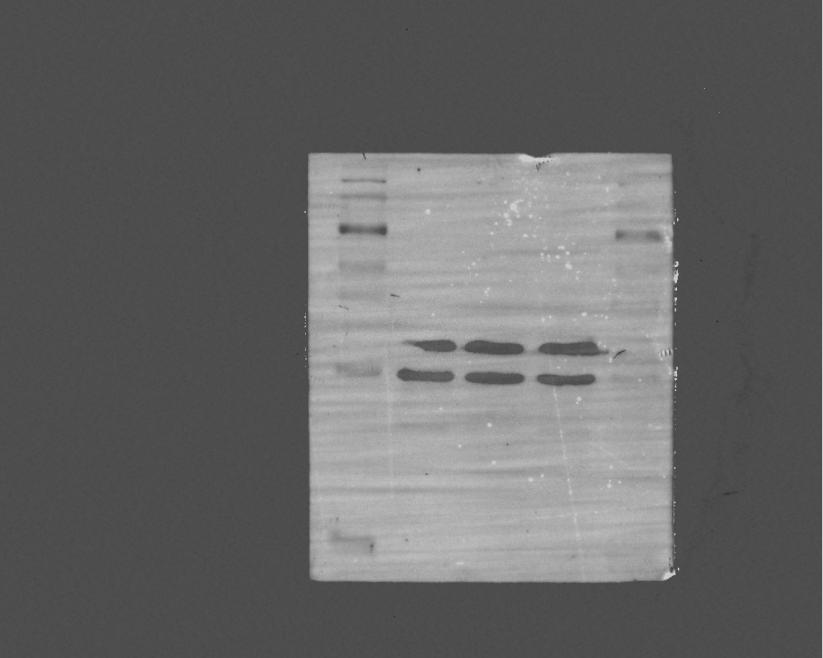


BAX(21KDa)

Figure 3f(Ishikawa)


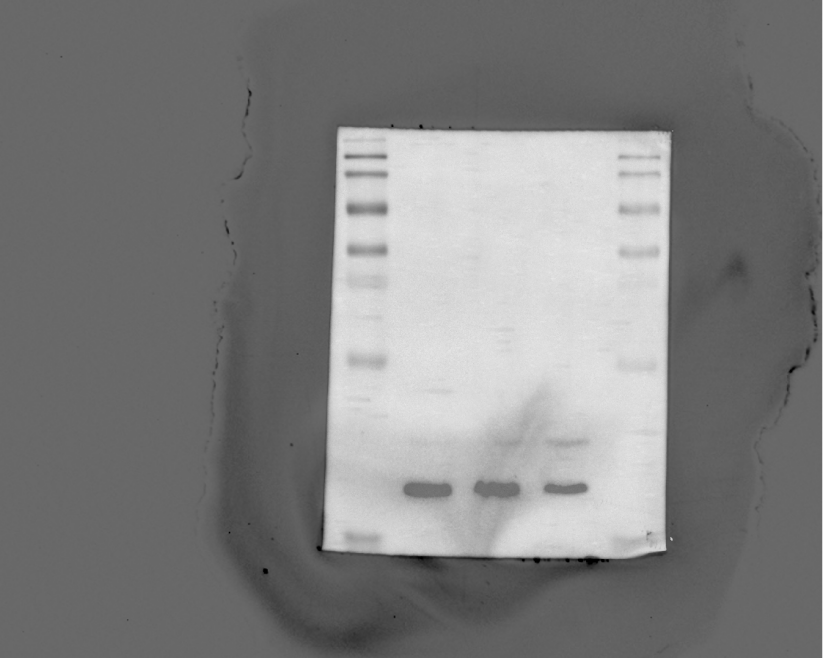


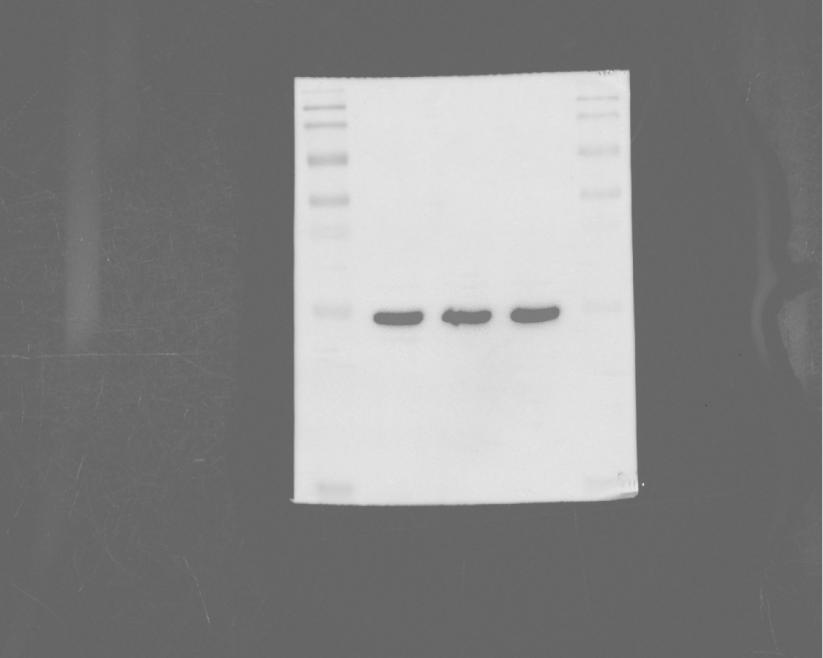


BAX(21KDa)

Figure 3f(SNGM)


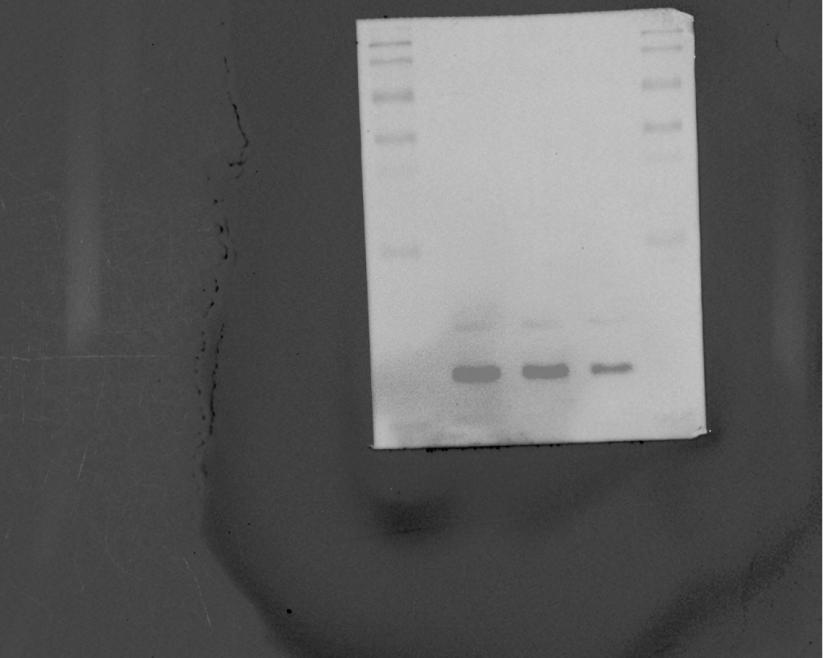


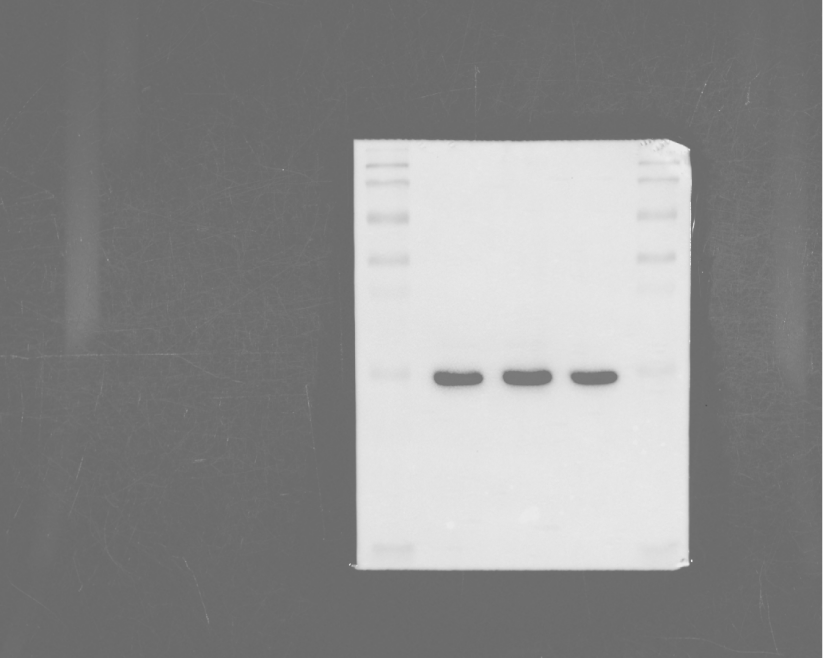


BCL- 2(28kDa)

Figure 3f(Ishikawa)


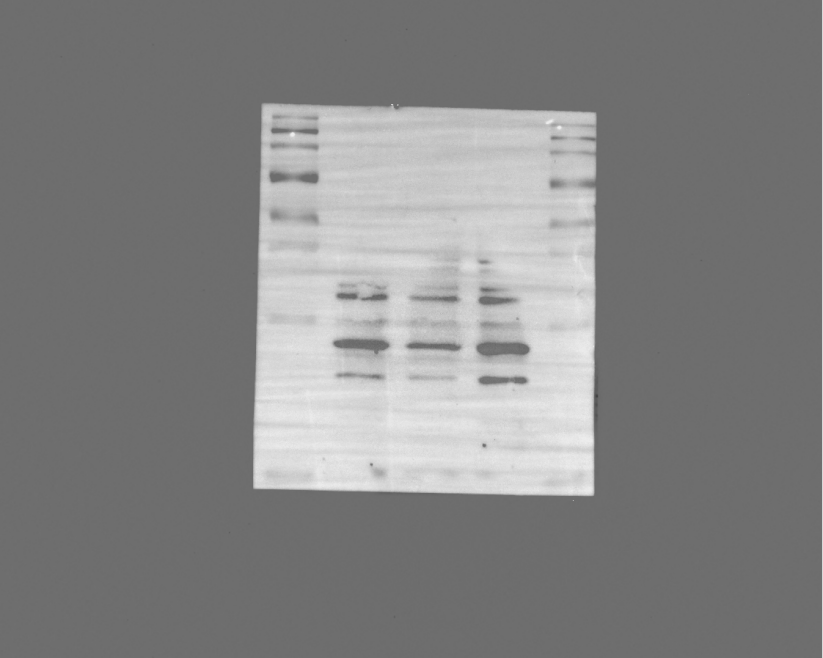


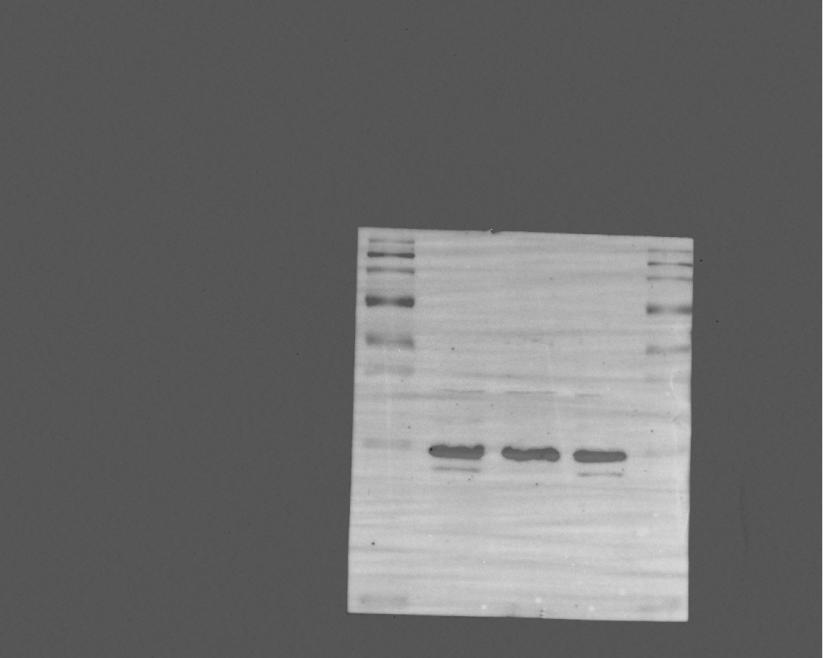


BCL- 2(28kDa)

Figure 3f(SNGM)


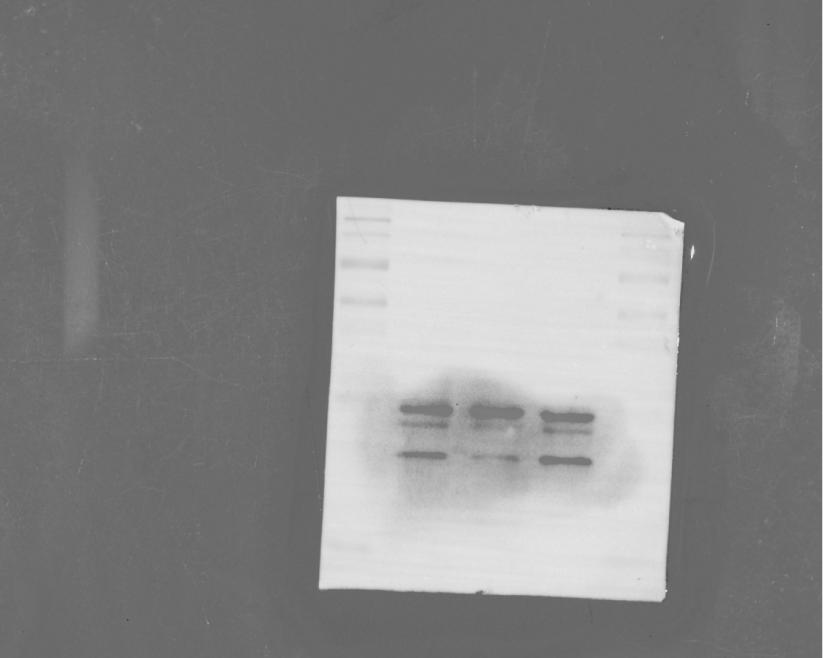


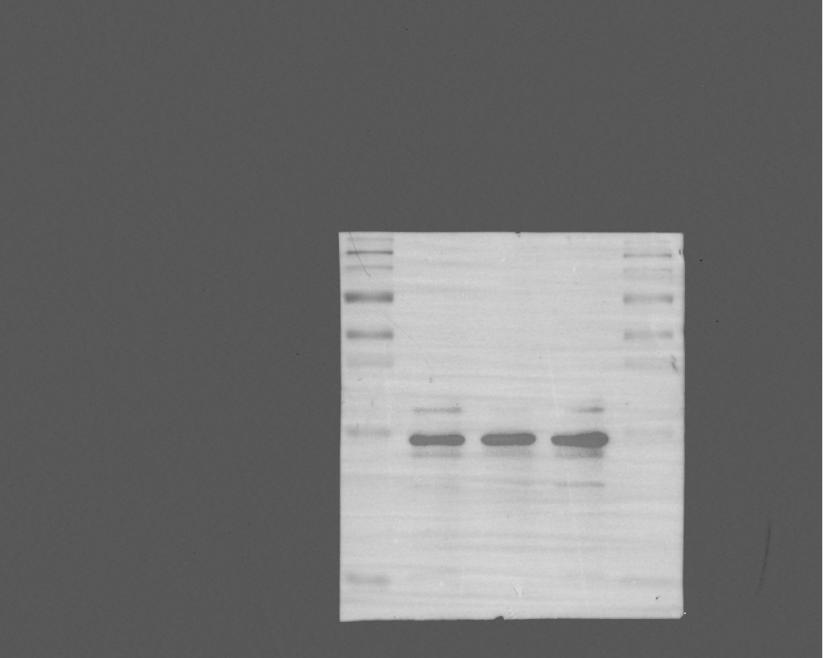


caspase3(35KDa)

Figure 3f(Ishikawa)


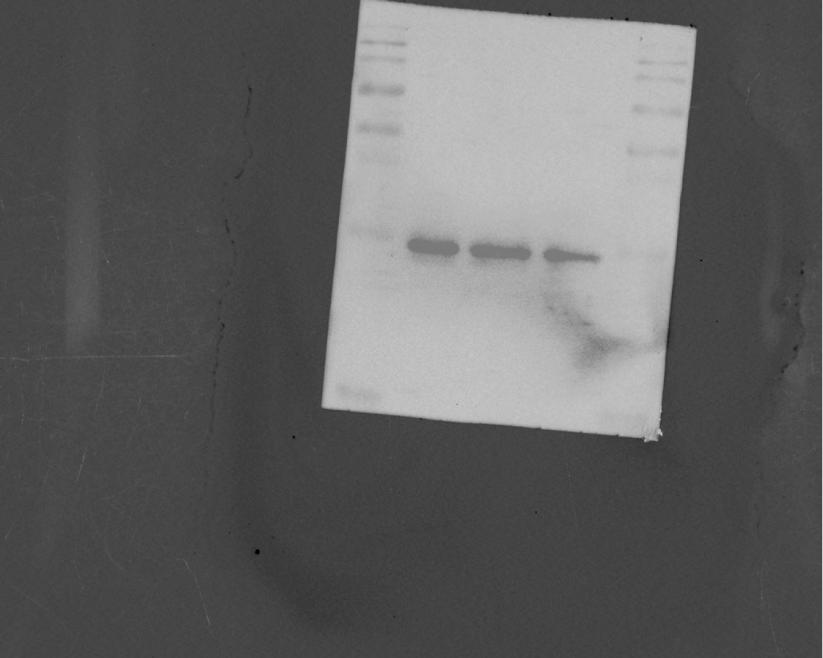


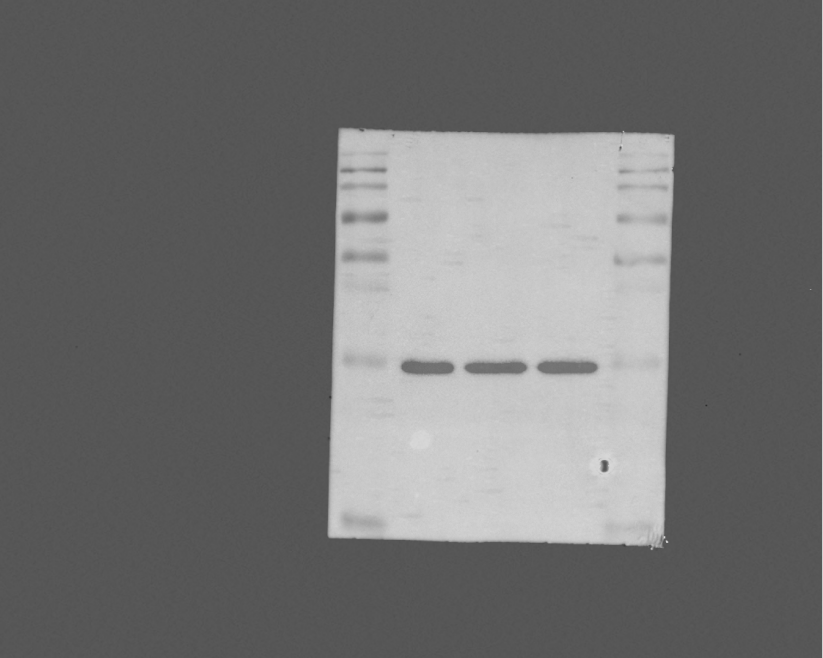


caspase3(35KDa)

Figure 3f(SNGM)


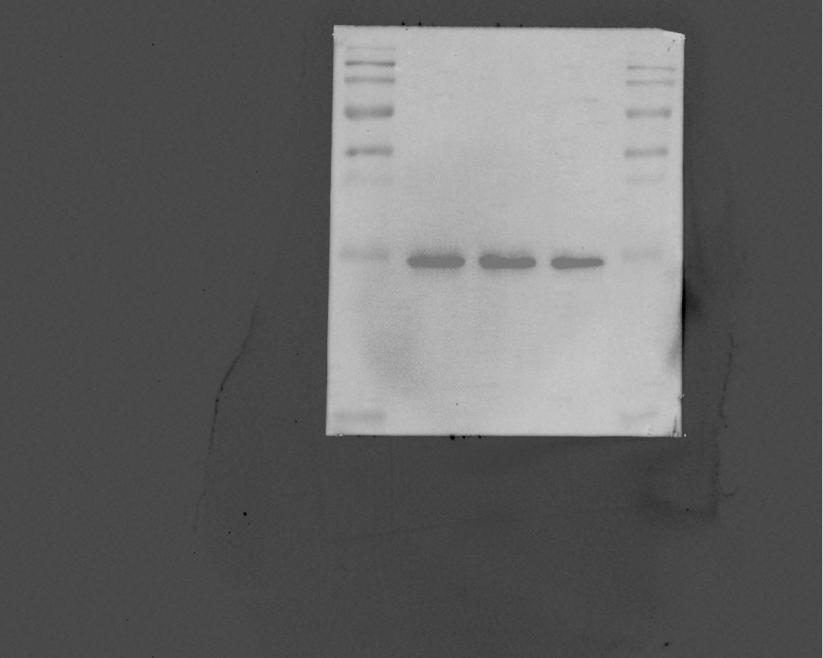


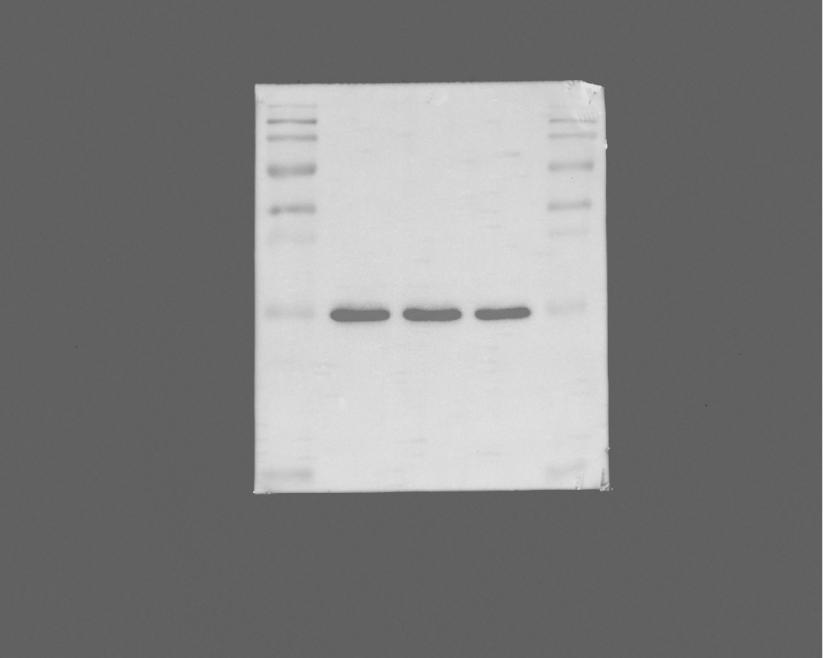

Supplement: Supplementary file 1 — Additional file 1. Raw data from western blotting. [file 12672_2024_937_MOESM1_ESM.docx]
